# Hub Genes in Stable QTLs Orchestrate the Accumulation of Cottonseed Oil in Upland Cotton via Catalyzing Key Steps of Lipid-Related Pathways

**DOI:** 10.3390/ijms242316595

**Published:** 2023-11-22

**Authors:** Beena Alam, Ruixian Liu, Juwu Gong, Junwen Li, Haoliang Yan, Qun Ge, Xianghui Xiao, Jingtao Pan, Haihong Shang, Yuzhen Shi, Youlu Yuan, Wankui Gong

**Affiliations:** 1 National Key Laboratory of Cotton Bio-Breeding and Integrated Utilization, Institute of Cotton Research, Chinese Academy of Agricultural Sciences, Anyang 455000, Chinashiyuzhen@caas.cn (Y.S.); 2 Zhengzhou Research Base, National Key Laboratory of Cotton Bio-Breeding and Integrated Utilization, Zhengzhou University, Zhengzhou 450001, China

**Keywords:** quantitative trait locus (QTL), kernel oil content (KOC), cottonseed, hub genes, *Gossypium hirsutum*

## Abstract

Upland cotton is the fifth-largest oil crop in the world, with an average supply of nearly 20% of vegetable oil production. Cottonseed oil is also an ideal alternative raw material to be efficiently converted into biodiesel. However, the improvement in kernel oil content (KOC) of cottonseed has not received sufficient attention from researchers for a long time, due to the fact that the main product of cotton planting is fiber. Previous studies have tagged QTLs and identified individual candidate genes that regulate KOC of cottonseed. The regulatory mechanism of oil metabolism and accumulation of cottonseed are still elusive. In the current study, two high-density genetic maps (HDGMs), which were constructed based on a recombinant inbred line (RIL) population consisting of 231 individuals, were used to identify KOC QTLs. A total of forty-three stable QTLs were detected via these two HDGM strategies. Bioinformatic analysis of all the genes harbored in the marker intervals of the stable QTLs revealed that a total of fifty-one genes were involved in the pathways related to lipid biosynthesis. Functional analysis via coexpression network and RNA-seq revealed that the hub genes in the co-expression network that also catalyze the key steps of fatty acid synthesis, lipid metabolism and oil body formation pathways (*ACX4*, *LACS4*, *KCR1*, and *SQD1*) could jointly orchestrate oil accumulation in cottonseed. This study will strengthen our understanding of oil metabolism and accumulation in cottonseed and contribute to KOC improvement in cottonseed in the future, enhancing the security and stability of worldwide food supply.

## 1. Introduction

Edible vegetable oils can not only be used as foods or feeds to alleviate global hunger and poverty but also as eco-friendly biofuels as alternatives to the increasingly scarce fossil fuels. *Gossypium* is the largest genus of the Malvaceae family [1,2]. Among the four domesticated species, upland cotton (*G*. *hirsutum*) is widely planted due to its wide adaptability and high yield, accounting for over 90% of the total cotton planting area. After cotton fiber, the first main product of cotton, which is ginned from the cottonseed, the second major product is cottonseed, which mainly consists of fuzz, hull and kernel. A cotton plant normally produces about 1.6 kg of seeds for every kilogram of lint produced. The cottonseed kernel accumulates 28.24–44.05% vegetable oil [1,3], which provides about 20% of worldwide oil product [2,4] and is recognized as the fifth-largest oil crop in the world, following soybean, palm, canola and sunflower [1]. The fatty acid (FA) composition of cottonseed oil makes it a vital source of vegetable oil, livestock feed and industrial bio-products, including lubricant oil and biodiesel [3,5,6,7]. The various unsaturation levels and carbon chain lengths of FAs can be used to define its nutritional and industrial values. Due to its flavor stability, it is also applied in a wide range of food processing [5]. Cottonseed oil is mainly accumulated in kernels of seeds and as the main product of cotton production is lint, the genetic regulatory mechanism of kernel oil content (KOC) accumulation and strategies for its improvement have been paid little attention. Some studies revealed that KOC is regulated in a complex quantitative genetic mechanism and significantly influenced by environmental parameters [7]. Molecular markers are crucial tools for genomic exploration, molecular description, and identification of phenotypic/genetic deviations. With the release of genome sequences of cotton species [8], plenty of reference genomes have been constructed [9,10]. These data have provided useful platforms for genetic marker development via various genotyping by sequencing (GBS) strategies, including specific locus amplified fragment sequencing (SLAF-seq), restriction site-associated DNA sequencing (RAD-seq) and microarray sequencing based on molecular hybridization [11]. Several high-density genetic maps (HDGMs) have been constructed using these markers and QTLs of KOC have thus been identified [5,7]. QTL mapping and candidate gene identification have been proven to be the basis of molecular marker-assisted selection (MAS), which has been confirmed to be a robust and operative breeding tool to improve the QTL-controlling traits [11]. However, in breeding practices, MAS is rarely used, mainly due to the lack of molecular markers closely linked to the target traits [12]. As far as the improvement in cottonseed KOC via MAS is concerned, it was estimated that the conversion of acetyl-CoA into seed oil directly requires at least 13 enzyme-dependent reactions [8]. This complex enzymatic reaction network makes it challenging to improve cottonseed KOC via conventional breeding strategies.

Recently, a natural mutant of FA desaturase-2 gene (*FAD2-1D*) was identified in accession GB-713 of *G*. *barbadense* and a functional link between the mutation and the increased oleic acid cottonseed oil was demonstrated [13]. In *G*. *hirsutum*, there are at least four different genes encoding *FAD2*, with *ghFAD2-1* playing a major role in the production of linoleic acid of cottonseed oil. The expression of *ghFAD2-1* is seed-specific and reaches its highest level at the middle maturity stage, between 25–35 days post-anthesis (dpa) [14,15]. Overexpression of this enzyme in cottonseed could increase the content of oleic acid [16]. In addition, double-bond introducing genes that turn oleic acid into linoleic acid play a key role in improving the oil quality via altering its FA composition from a perspective of health considerations. A class of desaturases, stearoyl-acyl carrier protein (ACP) desaturase (SAD), regulates the conversion of saturated FAs into unsaturated ones [17]. A previous study revealed that two *SADs* genes *GhSAD2* and *GhSAD4* in upland cotton are preferentially expressed in developing ovules [18]. Lysophosphatidic acid acyltransferase (LPAAT) was also reported to be involved in oil biosynthesis [7], catalyzing the rate-limiting step in the Kennedy pathway. Enhancing expression of Gh13LPAAT5 in Arabidopsis significantly increase the total oil accumulation in Arabidopsis seed [19]. *GhWRI1*, a transcription factor gene, was demonstrated to regulate oil accumulation in cottonseed. It may also participate in the responses of cotton to abiotic stresses [20]. These genes involved in cottonseed oil metabolism are mainly identified via linkage analysis [1,5,7], association analysis [21], or RNA expression analysis [22,23]. However, these studies still do not elucidate the mechanism by which the candidate genes regulate oil accumulation in cottonseed, especially how these individual genes form an interactive or co-expression network to perform their function.

In the current study, an RIL population consisting of 231 lines from a cross between a commercial cultivar with high fiber yield and low KOC of cottonseed, Lumianyan 28 (LMY28), and a cultivar with high fiber quality and high KOC, Xinluzao 24 (XLZ24), was investigated to answer the above question. Based on two HDGMs constructed via SLAF-seq genotyping and the Intl Cotton SNP Consortium_80k CHIP (CHIP) [24] in which we used both SLAF-seq genotyping technology and CHIP SNP marker set to genotype the RIL population. QTLs of KOC were identified and key candidate genes harbored in the QTLs were analyzed via RNA-seq data. Eventually, a co-expression network was identified, in which eight hub genes had dynamic expression during ovule development and cottonseed maturation. The hub genes *ACX4*, *LACS4*, *KCR1*, and *SQD1* located at the critical steps of both lipid biosynthesis and co-expression network suggest their functions in regulating oil accumulation in cottonseed. These results will help to better understand the genetic and molecular architecture of KOC of cottonseed, and will also provide useful sources for future research on KOC improvement projects.

## 2. Results

### 2.1. KOC Phenotypic Characteristics of the Parents and RIL Population

**Figure 1:**
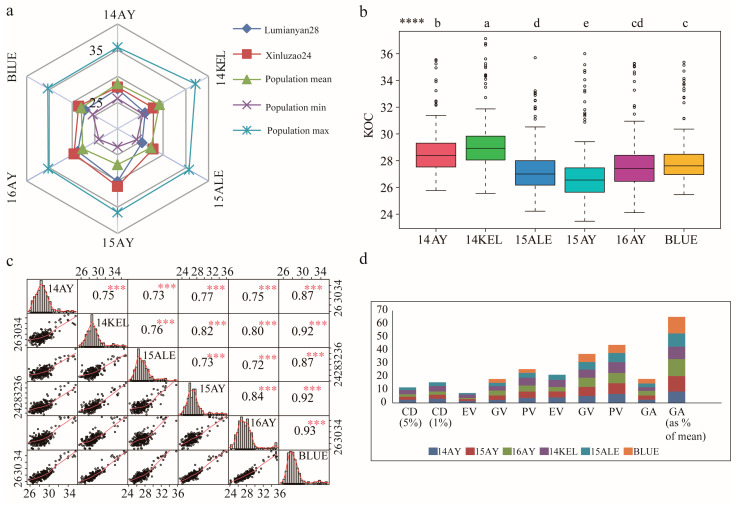
Phenotypic evaluation of kernel oil content (KOC) of cottonseed of the RILs and their parental lines across multiple environments. (**a**) Phenotypic performance of KOC of RILs, including highest, lowest and average values, and their parental lines. (**b**) Variance analysis of KOC of the RILs across six environments. (**c**) Scatterplots, distribution maps and correlation coefficients of KOC of RILs across different environments. (**d**) Genetic variability assessment of KOC of RILs across different environments. **Note:** population mean, mean value of KOC of RILs; population min, minimum value of KOC of RILs; population max, maximum value of KOC of RILs; 14AY, 2014 Anyang; 14KEL, 2014 Kuerle; 15ALE, 2015 Alaer; 15AY, 2015 Anyang; 16AY, 2016 Anyang; BLUE, the best linear unbiased estimate; CD, critical distance; EV, environmental variance; GV, genotypic variance; PV, phenotypic variance; ECV, environmental coefficient of variation; GCV, genotypic coefficient of variation; PCV, phenotypic coefficient of variation; GA, genetic advance. **** and a, b, c, d, e, indicate the significance of KOC phenotype differences between different environment at the *p* < 0.05 level in multiple comparisons; ***, indicates the correlation of KOC phenotypes between different environments reach a significant level of *p* < 0.001.

Descriptive statistics of KOC phenotypes of upland cottonseed of parents (LMY28 and XLZ24) and RIL population across six environments are presented in Table 1. Seeds of XLZ24 accumulated more KOC than LMY28 across all the environments and their differences reached *p* < 0.01 significance (Table 1, Figure 1a). The phenotypic distributions of KOC of the RILs in the Cotton Regions of the Yellow River Valley (YeRV) (namely 14AY, 15AY and 16AY) and of the Northwest Inland (NWI) (namely 14KEL and 15ALE) ranged from 24.21 to 37.13%. The mean values of the RILs in 14AY, 15AY, 16AY, 14KEL, 15ALE, and in BLUE were 28.60%, 26.86%, 27.7%, 29.20%, 27.31%, and 28.00% respectively. Likewise, the coefficients of variation (CV%) for KOC were 5.96%, 7.65%, 7.33%, 6.59%, 6.38%, and 6.19%, respectively (Table 1).

Wide-ranging variations in the KOC phenotypes of cottonseeds of the 231 RIL accessions were observed across six environments from 2014 to 2016. ANOVA revealed that the variances of KOC phenotypes of the population sourced from genotype reached great significance, implying reliability of further analysis in the current study (Table S2). In each environment, both positive and negative transgressive segregations (the observed phenotypic KOC values higher than that of XLZ24 or lower than that of LMY28, respectively) in the RILs were also observed (Figure 1b). The absolute values of skewness and kurtosis of KOC were less than one in most of the environments, indicating that KOCs of individuals in the population did not deviate and that they significantly fitted the normal distribution of typical quantitative traits (Figure 1c and Figure S1, Table S3). It was observed that the phenotypic performances of KOC across different environments were significantly correlated. The maximum correlation coefficient was identified between 16AY and the estimated BLUE (r = 0.93) (Figure 1c). The results also indicated that the KOC in 14KEL had comparatively lower correlation with those in other environments, with correlation coefficients ranging from 0.72 to 0.73 (Figure 1c). Strong broad-sense heritability was observed, which suggests a high contribution of genetic diversity to the phenotypic variation of upland cottonseed KOC under these environments (Figure 1d). All the statistical analysis proposed that cottonseed KOC traits in this research panel provided a good basis for further genetic analysis.

### 2.2. Hierarchical Cluster Analysis (HCA) and Discrimination of KOC Trait of the RILs

**Figure 2:**
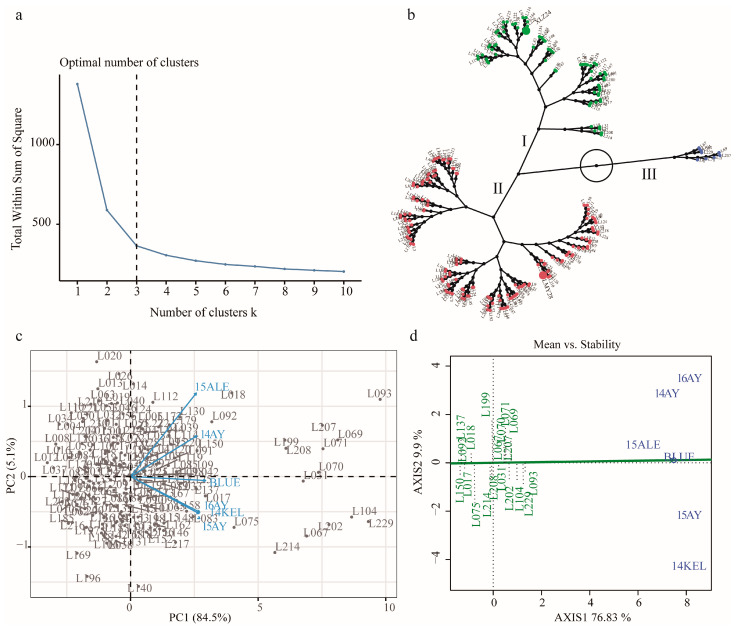
Discriminating analysis of KOC phenotypic performance of the RILs across multiple environments. (**a**) Analysis and determination of the optimal cluster number (k) of KOC of the RILs. (**b**) Clustering analysis of the RILs. (**c**) GGE Biplot analysis of KOC phenotypes of the RILs. (**d**) KOC stability analysis of high-KOC RILs (cluster 3) across multiple environments.

The individuals of the RILs underwent HCA based on their KOC performance across all environments, including their BLUE values. The results indicated that when the number of clusters k = 3, individuals of the RILs can be best discriminated based on their KOC level and phenotypic stability across the environments (Figure 2a and Figure S2). Cluster 1 and cluster 2, which were categorized as medium- and low-KOC clusters and into which the two parental lines XLZ24 and LMY28 fell, consisted of 94 and 123 individuals, respectively. Cluster 3 consisted of 19 high-KOC individuals, which had accumulated 29.99–35.36% of KOC in mature cottonseed (Figure 2b, Tables S3 and S4). The principal component (PC) analysis revealed that two PCs explained a total of 89.6% phenotypic variances, with PC1 84.5% and PC2 5.1%, respectively. The environmental vectors revealed that KOC phenotypes were positively correlated across different environments (Figure 2c). Phenotypic stability assessment of cluster 3 accessions via GGE biplot analysis revealed that these lines not only accumulated high KOC in mature cottonseeds but also that their accumulations had a high stability across different environments in the current study (Figure 2d).

### 2.3. QTL Mapping of KOC in RIL Population

**Figure 3:**
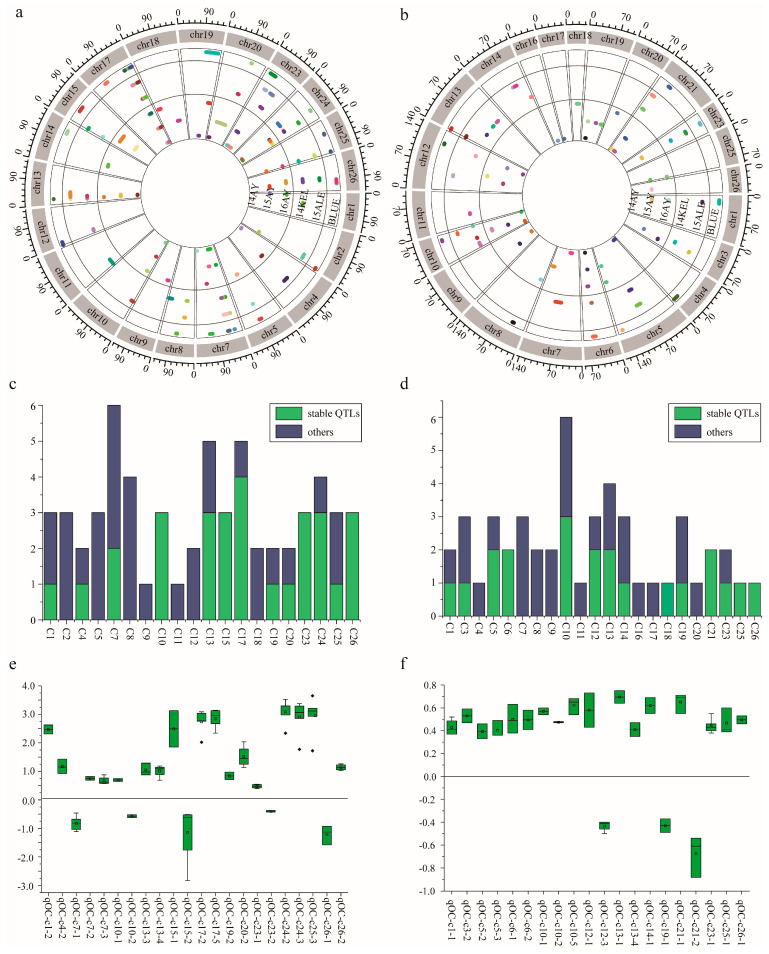
QTL identification via two high-density genetic maps (HDGMs) and their property analysis. (**a**) QTLs identified by HDGM of SLAF-seq strategy and their distribution across the genome. (**b**) QTLs identified by HDGM of CHIP strategy, and their distribution across the genome. (**c**) Stable QTLs identified by SLAF-seq strategy. (**d**) Stable QTLs identified by CHIP strategy. (**e**) Additive effect analysis of the stable QTLs identified by SLAF-seq strategy. (**f**) Additive effect analysis of the stable QTLs identified by CHIP strategy. **Note:** Colors represent different QTLs in each environment; The blue bars show the number of stable QTLs expressed in at least two environments and red bars indicate the number of other QTLs expressed in only one environment.

Based on the two HDGMs, which were constructed via SLAF-seq genotyping and CHIP genotyping strategies [24], respectively, a total of 105 KOC QTLs were identified, including 48 QTLs via CHIP (Figure 3a) and SLAF-seq 57 (Figure 3b) strategies. In sum, 43 QTLs were detected in at least two environments or by both strategies, which were regarded as stable ones (Figure 3c,d, Figures S3 and S4, Tables S5 and S6).

#### 2.3.1. QTLs Identified via SLAF-Seq Strategy

A total of 57 QTLs of KOC were identified by the composite interval mapping (CIM) algorithm on 21 chromosomes, except c3, c6, c16, c21 and c22 across the cotton genome (Figure 3a, Table S5). In these QTLs, 23 were stable ones, which explained 3.20–59.79% of the total phenotypic variance, with LOD values ranging from 1.74 to 20.62 (Table S6). Four stable QTLs, qOC^slaf^-chr7-1, qOC^slaf^-chr17-3, qOC^slaf^-chr24-3 and qOC^slaf^-chr25-3 were detected in all six environments, explaining 3.57% to 59.01% of the total observed phenotypic variance, with LOD scores of 2.06–20.62. Four stable QTLs, qOC^slaf^-chr17-2, qOC^slaf^-chr17-4, qOC^slaf^-chr17-5 and qOC^slaf^-chr24-5 were detected in five environments, explaining 41.42 to 60.50% of the observed phenotypic variance, with LOD values of 3.08–19.95. Six stable QTLs, namely, qOC^slaf^-chr17-3, qOC^slaf^-chr13-4, qOC^slaf^-chr15-2, qOC^slaf^-chr20-2, qOC^slaf^-chr23-1 and qOC^slaf^-chr26-2, were detected in four environments (Figure 3c, Table S5). Eighteen QTLs had positive additive effects, indicating that LMY28 contributed alleles in these loci that favor KOC accumulation in cottonseed, while the remaining five QTLs had negative additive effects, indicating that XLZ24 contributed favorable alleles for KOC accumulation. Ten stable QTLs explained >10% phenotypic variation, the favorable alleles of which are all from LMY28 (Figure 3e).

#### 2.3.2. QTLs Identified via CHIP Strategy

A total of 48 QTLs of KOC were identified by the CIM algorithm on 22 chromosomes, except c2, c15, c22 and c24 across the cotton genome (Figure 3b and Figure S3, Table S6). In these QTLs, 20 were stable ones, which explained 2.99–11.10% of the total phenotypic variance, with LOD values ranging from 1.69 to 5.47 (Table S6). In these stable QTLs, qOC^chip^-chr23-1 was detected in all six environments, explaining 3.36–6.97% of the total observed phenotypic variance (R^2^), with LOD scores of 2.01–4.00. OC^chip^-chr12-3 and qOC^chip^-chr1-1 were detected in five and four environments, respectively, explaining 3.61–5.45% and 4.01–4.89% of the observed phenotypic variance with LOD values of 2.04–3.05 and 2.06–2.59, respectively. Six stable QTLs, namely qOC^chip^-chr5-3, qOC^chip^-chr6-1, qOC^chip^-chr10-5, qOC^chip^-chr21-1, qOC^chip^-chr21-2 and qOC^chip^-chr25-1 were detected in three environments (Figure 3d, Table S6). Seventeen QTLs had positive additive effects, indicating that LMY28 contributed alleles in these loci that favor KOC accumulation in cottonseed, while the remaining three QTLs had negative additive effects, indicating that XLZ24 contributed favorable alleles for KOC accumulation. Three QTLs, namely, qOC-chr6-1, qOC^chip^-chr21-1 and qOC^chip^-chr21-2, explained >10% of the phenotypic variation; the favorable alleles of the former two were from LMY28 and that of the latter one from XLZ24 (Figure 3f).

### 2.4. Candidate Gene Identification and Functional Annotation

**Figure 4:**
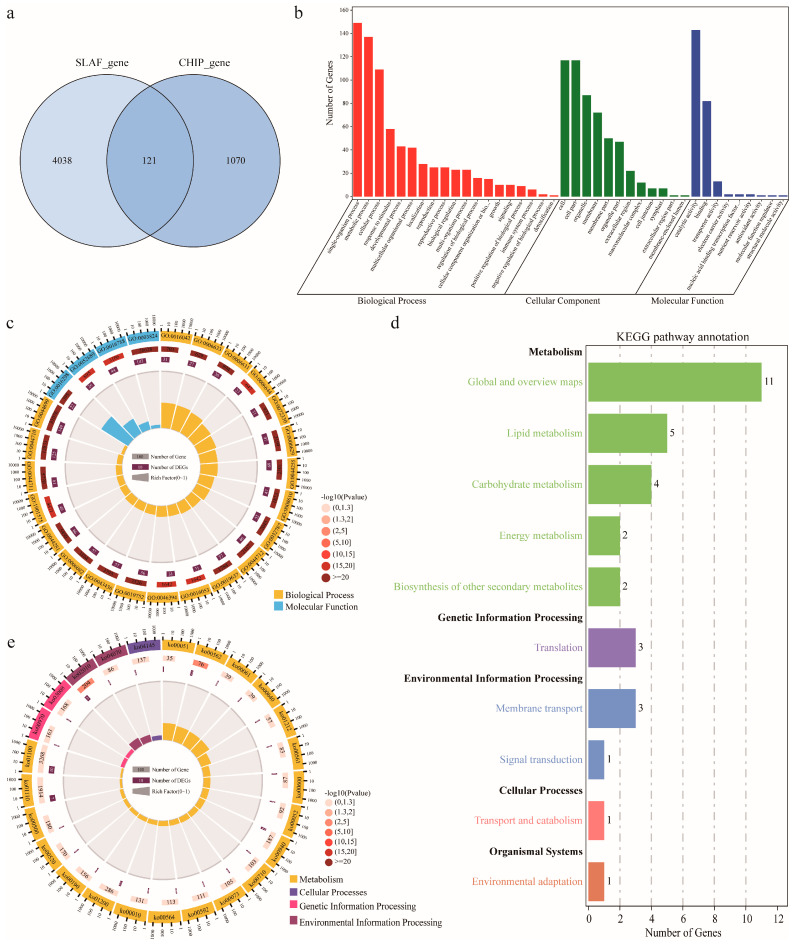
Bioinformatic analysis of the genes harbored in the stable QTLs identified by both high-density genetic maps (HDGMs). (**a**) Total number of the genes by the two HDGMs. (**b**) Gene Ontology enrichment analysis of the genes. (**c**) The first twenty terms significantly enriched in GO analysis. (**d**) Kyoto Encyclopedia of Genes and Genomes (KEGG) pathway annotations of the genes. (**e**) The first twenty pathways significantly enriched in KEGG pathway annotations.

Based on the physical position of the markers [9], a total of 5229 genes were obtained within the confidence marker interval of stable QTLs identified on the two HDGMs, of which 4159 genes were obtained on the HDGM constructed via SLAF-seq strategy and 1191 genes on the one via CHIP strategy. Of these 5229 gene, 121 genes were obtained simultaneously from both of the two maps (Figure 4a). When these gene were aligned to the Arabidopsis Acyl-Lipid Metabolism database (http://aralip.plantbiology.msu.edu/pathways/pathways, accessed on 23 December 2022) [25], a total of 162 genes including 134 coding genes and 1 TFs were identified to have homologues in the database (Table S7). These 162 genes might participate in lipid metabolism and were used for further functional annotation. GO term enrichment revealed that the genes were mainly enriched in the categories of biological process, cellular component and molecular function (Figure 4b,c). The first 20 significantly enriched GO terms were mainly accumulated in those of biological process and molecular function (Figure 4c). Kyoto Encyclopedia of Genes and Genomes (KEGG) analysis of these genes revealed that their functional annotations were mainly enriched in the pathways of metabolism, genetic information processing, and cellular processes (Figure 4d,e). A total of 51 genes were identified to be involved in the pathways related to lipid biosynthesis.

**Figure 5:**
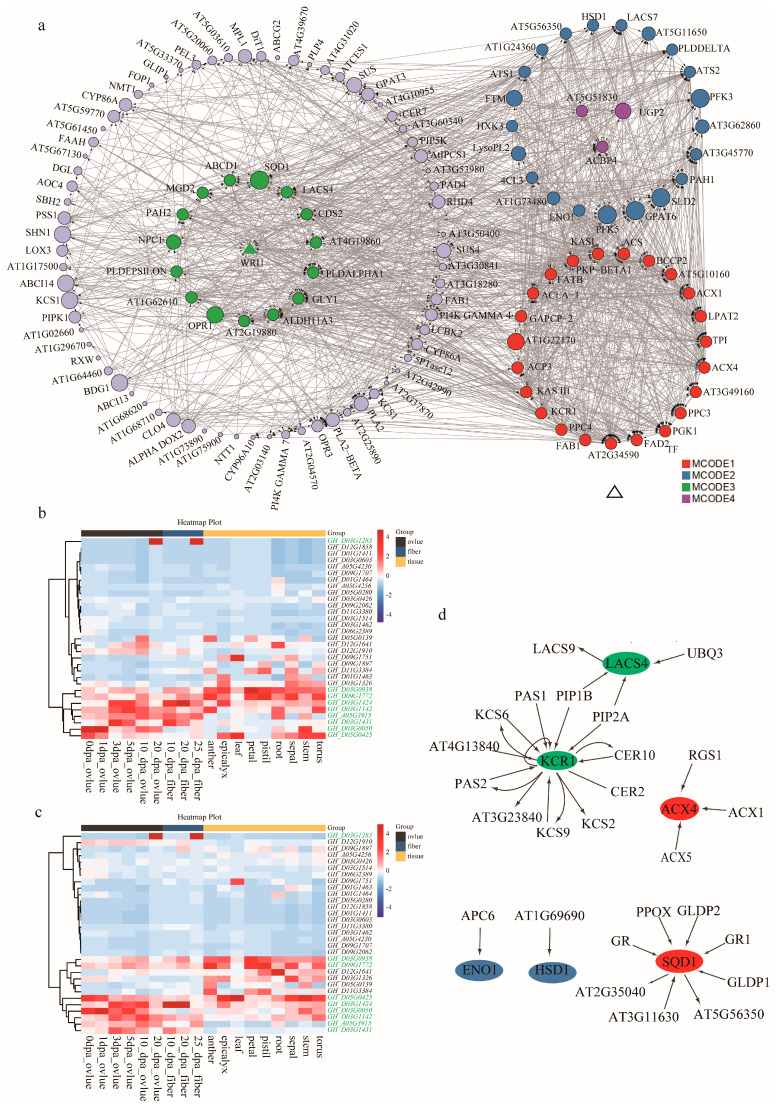
Co-expression network construction and hub gene expression verification during ovule development. (**a**) The network consists of four MCODEs, which are involved in pathways relating carbon metabolism, FAs and lipid biosynthesis, glycolysis. (**b**,**c**) Heatmaps of dynamic expression profiles of the hub genes in various organs and developing stages of upland cotton cultivars TM-1 and Hai7124, respectively. (**d**) Interactions of the hub genes that catalyze the key steps of lipid metabolic pathways.

To further capture how the possible candidate genes orchestrate KOC accumulation, the co-expression network among the proteins the 162 genes was explored via the Metascape database. The network identified four core MCODE components, confining a total of 63 hub genes, as well as complex interactions among genes within and between the components (Figure 5a). MCODE 1 consisted of 23 hub genes which were mainly involved in the metabolism of monocarboxylic, and FAs—carbon metabolism. MCODE 2 consisted of 21 hub genes that were mainly involved in monocarboxylic acid, organic phosphorus, and ADP metabolism. MCODE 3 consisted of 16 hub genes that were mainly involved in lipid biosynthesis, lipid metabolism, and signal transduction. MCODE 4 consisted of three hub genes that were mainly involved in glycolysis (Figure 5a).

### 2.5. Candidate Gene Expression Verification during Ovule Development

Further expression verification of these hub genes via RNA-seq data of different tissues/organs and different developmental stages of ovules of *Gossypium* germplasms TM-1 and Hai7124 revealed that eight hub genes have dynamic relative expression profiling during ovule development in both TM-1 and Hai7124 (Table 2, Figure 5b,c and Figure S5). *GH_A05G3915* had a medium to high expression in developing ovules of TM-1 from 3 dpa to 20 dpa, while in those of Hai7124 only from 10 to 20 dpa (Figure 5b,c). *GH_D03G0050* and *GH_D05G0425* had a high expression in developing ovules at stages of 3 and 5 dpa of Hai7124 than those of TM-1. *GH_D03G0050* and *GH_A05G3915* had a higher expression in developing ovules of TM-1 than of H7124 at 20 dpa. *GH_D09G1772* had a higher expression in developing ovules of TM-1 than of Hai7124 at 10 dpa. *GH_D03G1283* had a higher expression in 20 dpa developing ovules and 25 dpa developing fibers of both TM-1 and Hai7124. Both *GH_D03G0050* and *GH_D05G0425* had a high expression in ovules of both germplasms TM-1 and Hai7124 from 0 to 1 dpa, however, the expression of *GH_D03G0050* was more highly induced in TM-1 ovules than in H7124 ovules, and in the latter, its expression may last longer. *GH_D03G1424*, *GH_D03G1431* and *GH_D03G1142* had high expression profiles in developing ovules from 3 to 10 dpa; however, their expression in TM-1 was higher in Hai7124 (Figure 5b,c). These dynamically expressed hub genes that catalyze the key steps in the MCODE networks may take responsible roles in KOC formation (Figure 5d).

## 3. Discussion

### 3.1. Plant Materials and Their Cottonseed KOC Performance across Various Environments

The RIL population of this study, together with their two parental lines, LMY28 and XLZ24, were planted in six different geographical and chronological environments. KOCs of LMY28 and XLZ24 were different across the different environments, especially in 15AY and 15ALE (Table 1). The diverse variations of climate factors in these experimental locations and across different years must have contributed to the KOC phenotypic variations of the materials used for QTL analysis. Therefore, planting in multiple environments expanded the phenotypic variations of these materials. Strong broad-sense heritability of the experimental materials under multiple environments suggests that the cottonseed KOC has a highly diverse genetic composition. The stability and correlation of KOC across different environments provides a good basis for further genetic analysis. Statistical analysis showed that the KOC of cottonseed in this research panel did not deviate and showed a roughly normal distribution, which confirmed that KOC was controlled by multiple genes and could be analyzed through linkage analysis [9]. Hierarchical cluster analysis (HCA) is essentially helpful to classify the relationship of the experimental materials according to the similarity or dissimilarity and the relationship of the characteristics of the variables [27]. HCA distinguished groups of individuals which maintained similar behavior patterns and further validated the PCA results [28]. The current study finally observed optimally three clustering groups (k = 3) of KOC phenotypes of the RIL population (Figure 2a,b). In phenotypic evaluations, the KOC of XLZ24 was higher than that of LMY28 (Figure 1a, Table 1), which is consistent with the fact that XLZ24 contributed favorable alleles of the majority of stable QTLs for oil accumulation in cottonseeds. However, the inferior parent LMY28 also contributed favorable alleles of some stable QTLs to enhance the trait value (Figure 3e,f). The recombination of the favorable alleles of these QTLs contributed to the transgressive segregation in progeny RILs and the relative consistency of the high KOC lines across different environments (cluster 3, Figure 2b). In previous QTL mapping reports, it was also detected that the beneficial alleles of QTLs of a target trait might come from different parental lines, that is to say, the beneficial alleles of the two parental lines co-determine the formation of the target trait in the population [1,29]. These consistent observations show that it is effective to improve cottonseed KOC through genetic improvement, and these high-KOC lines can play an effective role in future breeding projects to improve KOC of cottonseed. Due to the unique nature of cotton, its fiber quality plays a decisive role in cotton breeding research. In the current study, the materials used are derived from the varieties in practical cotton production [24]. Therefore, these high-KOC lines will have practical application value and can be applied to the simultaneous improvement of both fiber quality and KOC of cottonseed.

### 3.2. HDGM and QTL Mapping of Cottonseed KOC

Increasing the number of markers could improve the accuracy of QTL analysis and thereby identifying the potential candidate genes in these functional genome loci. The application of AFLP, RFLP, and SSR markers in QTL identification of oil content has previously been limited, resulting in inaccurate QTL region identification [9,21]. However, some studies have tackled the candidate genes regulating TAG accumulation in cottonseed via QTL analysis or GWAS [7,23,30]. In the current study, two HDGMs were applied to the KOC QTL analysis of the RIL population, a total of 43 stable QTLs (23 from SLAF-sequence and 20 from CHIP strategies) were thus detected. To confirm the constancy and reliability of the QTLs detected from the two HDGMs, we compared the KOC-QTLs with those published previously from linkage analysis of segregating populations with different genetic backgrounds or GWAS of natural accession panels. Five KOC-QTLs qOC^slaf^-c17-3, qOC^slaf^-c23-1, qOC^chip^-c5-3, qOC^chip^-c6-1 and qOC^chip^-c23-1 were found to be overlapped with confidence intervals of published QTLs identified by other populations [7,21,31,32]. These QTLs are highly consistent and can be used for further marker assisted selection analysis. More interestingly, the current HDGMs based on SNPs had better resolution than the other maps used in previous reported studies. These results further provide a reliable roadmap for tracking the casual genes underlying KOC.

### 3.3. Candidate Genes Identification and Network Analysis

**Figure 6:**
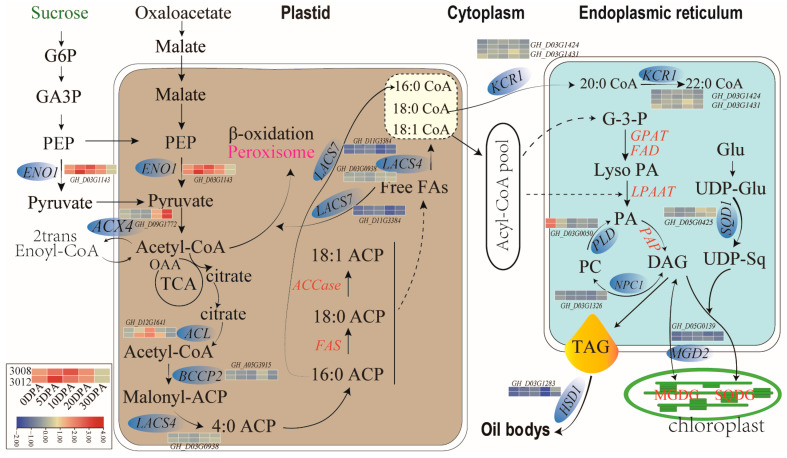
Hub genes in the expression network that have dynamic expressions in developing ovules and the catalytic steps of these hub genes in the pathways related to oil biosynthesis and oil body formation.

In plants, triacylglycerols (TAGs, esters of glycerol and FAs) are stored in oil bodies [33], which are dynamic spherical cytoplasmic organelles with 0.5–2.0 μm-diameter structures that contain the TAG matrix and its surrounding monolayer of phospholipids and oleosins [33,34]. They are integral to cell metabolism, with an effective lipids-turnover equilibrium of biogenesis and consumption [35,36]. During seed development, oil bodies are generated through the budding process of the smooth endoplasmic reticulum (ER), while TAGs and oleosins are produced and assembled [36]. Oleosins are 15–24 kDa alkaline proteins present in a variety of plant species. The structure of oleosins features in a C-terminal amphipathic α-helical domain, a central hydrophobic anti-parallel β-strand region, and an N-terminal amphipathic domain. It is reported that central hydrophobic domain anchors oleosin to oil bodies, while their N- and C-terminal amphipathic domains provide steric hindrance and electronegative repulsion to stabilize these lipid-storage organelles. The oxidation of FAs releases twice as much energy as the oxidation of carbohydrates or proteins, resulting in a significant reduction in carbon demand for energy storage in the form of FAs under the same storage conditions. Thus, TAGs represent a highly efficient form of energy storage. In seeds, the accumulation and stores of TAGs allow for enhanced germination and seedling growth until seedling photosynthesis is established [37]. Previous studies have tackled genes regulating TAG biosynthesis and oil body assembly [7]; however, they focused only on individual gene hunting through their specific strategies [38,39,40,41]. In the current study, we reported a co-expression network of candidate genes in the stable QTLs (Figure 5a). Expression analysis revealed that some hub genes in the network exhibited dynamic expressions during ovule development (Table 2), which strongly inferred that the interactions among these hub gene members in the network may jointly regulate ovule development and thereby determining the final accumulation of TAG in cottonseed. The hub genes, *ENO1* (*phosphoenolpyruvate enolase 1*, *GH_D03G1142*) [42], *ACX4* (*acyl-CoA oxidase-4*, *GH_D09G1772*) [43,44], *BCCP2* (*biotin carboxyl carrier protein 2*, *GH_A05G3915*) [23,41], *LACS4* (*long-chain acyl-CoA synthetase 4*, *GH_D03G0938*) [5,7,45,46] and *KCR1* (*ketoacyl CoA reductase 1*, *GH_D03G1424/GH_D03G1431*) [43] are involved in the steps of the pathways of FA biosynthesis and elongation, and the hub genes, *PLDδ* (*Phospholipase Dδ*, *GH_D03G0050*) [38], *SQD1* (*UDP-sulfoquinovose synthase 1*, *GH_D05G0425*) [47] and *HSD1* (*hydroxysteroid dehydrogenase 1*, *GH_D03G1283*) [48] are involved in TAG biosynthesis and oil body assembly (Figure 6). The hub genes *ACX4*, *LACS4*, *KCR1* and *SQD1* located at the key steps in the network (Figure 5d). *ACX4* encodes a peroxisomal acyl-CoA oxidase catalyzing b-oxidation of short-chain FAs, the conversion of Acyl-CoA to 2trans-Enoyl-CoA [43]. *ACX4* has been demonstrated to be the major enzyme that catalyzes short-chain substrate reactions [49]. The current study detected a dynamic increase in *ACX4* expression during ovule development, which is also consistent with previous observation on increased FA metabolism during embryonic development [44]. Very-long-chain FAs (VLCFAs), is demonstrated to play crucial roles in plant structure, physiology, and signaling. In upland cotton, modulating VLCFA biosynthesis during fiber development was demonstrated to regulate cotton fiber elongation [50]. *LACS4* was shown to provide CoA-activated VLCFAs (VLCFA-CoAs) to the pathways of FA elongation. *KCR1* functionally act on a membrane-bound FA elongation system, which multiple proteins might be involved [51]. A recent study demonstrated that KCR1 interacts with ketoacyl-CoA synthase (KCS) to form a FA elongase complex to catalyze biosynthesis of VLCFAs [52]. Our results suggest that *ACX4*, *LACS4* and *KCR1* might jointly catalyze a metabolism cascade of FA elongation and VLCFA synthesis (Figure 6), which further modulates oil accumulation in plant seeds [53]. *SQD1* was initially confirmed to mediate the biosynthesis of the sulfoquinovosyl headgroup of plant sulfolipids, catalyzing the transfer of SO_3_^−^ to UDP-glucose [54]. In higher plant, sulfoquinovosyl diacylglycerol, a sulfolipid, is one of the four characteristic lipids present in chloroplast membranes [47], which is required for full maintenance of activity and stability of photosystem II [55]. Our results imply that the expression of *SQD1* is highly correlated to oil accumulation in cottonseed. How exactly SQD1 regulates the accumulation of oil in seeds still remains open to discussion; however, previous findings provided some valuable hints for this issue. A more recent study revealed that a gene, *LPPϵ1*, the expression of which was localized at the chloroplast outer envelope, could regulate ER lipid metabolism via interaction with the gene, *LPPα2*, which were expressed at ER system [56]. The correlation between dynamic homeostasis of lipid in oil body and plant responses to abiotic stress has been well established [57]. Biosynthesis of sulfoquinovosyl diacylglycerol (SQDG) coded by *SQD1* as a surrogate lipid for phosphatidylglycerol (PG) in chloroplast under phosphate deficiency [17] plays a role in abiotic stresses. Studies have revealed that genes with dynamic expression during plant embryo development and maturation are also active in plant responses to abiotic stresses [7,9,58]. In the abiotic stresses, including salt, drought, and freezing, a common phenomenon is the deficient availability of plant water status caused by external environmental factors [59]. The development and maturation of embryos may also undergo a process from sufficient to limited water supply caused by intrinsic physiological status of plant. These findings infer that the action mechanism of *SQD1* in seed oil accumulation might share a similarity to its role in regulating lipid dynamic homeostasis in plant responses to abiotic stresses. Interestingly, the oil body protein gene, *HSD1*, identified in the current study, is not involved in FA biosynthesis or TAG assembly (Figure 6). Studies have demonstrated that HSDs are minor components of oil body in oilseed crops [60,61] and that it is involved in lipid homeostasis [62] during embryo development, seed maturation, and plant responses to abiotic stresses [63].

However, oil biosynthesis during embryo development is a complex and hierarchical biological process that is regulated by some complex gene co-expression networks [64]. In *Arabidopsis thaliana*, metabolic pathways associated with the biosynthesis and degradation of acyl-lipids have been summarized and most of the genes have been described [25]. Single gene mutations may affect the expression of genes involved in the common metabolic network process. Even though, any modification in oil biosynthesis controlling enzymes induced the alteration in TAG synthesis and even in oil yield [65]. Therefore, further identification of the network is necessary for the rapid capture of causal candidate genes. Here, the screening of potential candidate genes based on KOC-QTLs provides more information to eliminate the interference from a perspective of competition mechanism and metabolism [66]. The current study provided useful information for us to understand the intricate regulatory mechanism of oil accumulation process and provided a foundation for further identification of the causative loci using linkage and transcriptome analyses.

## 4. Materials and Methods

### 4.1. Plant Materials and Field Evaluation

Two upland cotton (*G. hirsutum*) field cultivars, LMY28 and XLZ24, and the RIL population consisting of 231 lines derived from a cross of LMY28×XLZ24 were used as experimental plant materials in this study. The procedure of the development of RIL population and the characteristics of two parental cultivars were detailed previously [24]. Briefly the cross LMY28×XLZ24 was made in 2008 summer season in the farm of the Institute of Cotton Research of Chinese Academy of Agricultural Sciences, Anyang, Henan province, China. The RIL population was reached till F_6:8_ generation. The experimental materials were evaluated in field conditions across five different environments, including in Anyang, Henan province in 2014, 2015, and 2016 (designated as 14AY, 15AY and 16AY), in Kuerle in 2014 and in Alaer in 2015 in Xinjiang (designated as 14KEL and 15ALE respectively) [24]. The phenotypes of the target trait (KOC) of the experimental materials were evaluated after cottonseeds were ginned and husked. The best linear unbiased estimate (BLUE) of each line across the five environments, a theoretical value estimated by QTL ICIMapping 4.2 software [7], was regarded as the phenotype of another environment, which made a total of six different environments in the study. The field experiments were conducted in complete randomized block design with two biological replications in each environmental condition from 2014 to 2016.

### 4.2. Phenotypic Trait Evaluation

In harvest season, seed cotton of 50 typical bolls from each line was harvested. After dried, the seed cotton was ginned and the cottonseeds were delinted with dilute sulfuric acid solution. The delinted cottonseeds were dried and husked. The cottonseed kernels were stored at room temperature till KOC was evaluated. KOC was evaluated using the Niumag Imaging and Analyzing System (NMI20-Analyst, Shanghai, China), following a previously described protocol [5] with minor modifications.

### 4.3. Data Analysis

Descriptive statistics including the mean value, standard deviation, skewness and kurtosis, ANOVA of KOC in the experimental materials were performed. The recorded data of KOC traits were also subjected to evaluate descriptive statistics, ANOVA, correlation, principal component analysis (PCA), hierarchical cluster analysis (HCA), heat mapping and heritability (broad sense), critical distance in 5% and in 1%, phenotypic variance (PV), genotypic variance (GV), environmental variance (EV) and their coefficient of variation such as PCV, GCV and ECV, genetic advance (GA) and genetic advance as percentage of mean, in order to determine the significant genetic diversity among accessions. All the analyses were computed with Microsoft Excel 2016, IBM SPSS Statistics v. 25 (https://www.ibm.com/analytics/spss-statistics-software, accessed on 16 August 2022) and R-Studio v. 1.2.5001 (https://rstudio.com, accessed on 16 August 2022).

### 4.4. Linkage Mapping and QTL Identification

Two HDGMs were applied to the KOC QTL mapping of the RIL population. One HDGM was based on CHIP strategy, which contains 4851 molecular markers, including 122 SSR and 2719 SNP ones, with a total coverage of 2477.99 cM of *G*. *hirsutum* genome and an average marker interval of 0.51 cM between adjacent markers [24]. The other was based on the SLAF-seq strategy, which contains 4366 SNP markers with a total coverage of 3826.92 cM of *G*. *hirsutum* genome and an average marker interval of 0.73 cM (Liu et al., unpublished data). The brief depiction of the HDGMs of the RIL population is detailed in Table S1.

The CIM algorithms of Windows QTL Cartographer 2.5 software [67] with a mapping step of 1.0 cM and five control markers [68] was employed to detect the significance of a QTL existence in each individual environment. In the parameter setting, the threshold value of the logarithm of odds (LOD) was calculated with 1000 permutations (n = 1000) at the 0.05 significance level (*p* < 0.05). LOD score between 2.0 and permutation test LOD threshold were used to declare a suggestive QTL. QTLs identified in different environments that had fully or partially overlapping confidence intervals were regarded as the same QTL. The QTL detected in at least two environments was regarded as a potential stable one. Positive additive effect of a QTL means that its favorable allele was contributed by XLZ24 while negative additive effect means that its favorable allele contributed by LMY28. Stable QTLs were compared with the Cotton QTL database (https://www.cottonqtldb.org, accessed on 10 September 2022) [7] to determine whether they were previously identified or regarded as a new one. If the markers in the current study shared the same or overlapped physical position with those in the Cotton QTL database, then QTLs were regarded as previously published; otherwise, they were considered as new ones.

### 4.5. QTL Nomenclature

Nomenclature of QTL was designated following Sun’s description [69]. The rule of QTL nomenclature starts with the letter “q” followed by the abbreviation of the trait, the number of chromosome where the QTL was identified, and the QTL serial number on that chromosome, and these elements are separated by the symbol “-” (for example, qKOC-c06-2 represents the second QTL of KOC on chromosome 6).

Candidate Gene Identification and Functional Annotation

The markers flanking the confidence intervals of the stable QTLs were selected to identify the candidate genes. These markers were aligned back to the physical position of upland cotton genome database (www.cottonfgd.org, accessed on 25 October 2022). Based on the position of these flanking markers, all the genes within the confidence interval were selected to perform candidate gene identification. For some of the QTLs with a large confidence interval, if the position of one marker flanking the confidence interval was too far from that of the nearest marker harbored in that confidence interval, the region between these two markers was excluded from the candidate gene identification. All the selected genes were categorized through the gene ontology (GO) analysis using agriGO 2.0 software, and the pathways correlated to the candidate genes were discovered by the Kyoto Encyclopedia of Genes and Genomes (KEGG) analysis using KOBAS 3.0 [7]. The pathways which related to oil and FAs biosynthesis were considered as the enriched terms (for GO) and enriched functional pathways (for KEGG). Then, identified genes were compared with the transcriptomic database available online at functional cotton genome database (www.cottonfgd.org, accessed on 25 October 2022). Finally, the *G. hirsutum* gene expression database (ZJU Assembly) was used to find the expression of the genes of candidate regions in relevant tissues.

### 4.6. Differential Expression Profiling and Construction of Co-Expression Network of Candidate Genes

All genes in the confidence interval of stable QTLs and their corresponding protein sequences were blasted against the Arabidopsis database (https://www.arabidopsis.org/, accessed on 25 October 2022). Cotton expression profile data were downloaded from the database (https://cotton.zju.edu.cn/, accessed on 25 October 2022). The database contains FPKM values of developing ovules at 0, 1, 3, 5, 10, and 20 dpa of “TM-1”, a standard upland cotton line with a low KOC of 30.82%, and “Hai7124”, a cultivar with a high KOC of 35.31%. The TM-1 database was used to analyze the expression profiles of candidate genes in KOC QTLs, and the Hai7124 database was used to compare the differential expression of these genes during cotton ovule development between high and low KOC materials.

The Metascape database of Cytoscape 3.9.0 was used to conduct and visualize the regulatory co-expression network among the candidate genes encoding lipid biosynthesis and KOC related proteins. If the network contained 3–500 proteins, then the molecular complexity detection (MCODE) algorithm was used to identify the network components with density of node–node connections. For each MCODE component, pathway and process enrichment analyses were performed independently. The purpose of making this network was to elaborate the correlation among the genes encoding similar function performing proteins, especially the ones related to KOC of upland cotton [7].

## 5. Conclusions

The current study has identified 43 stable QTLs via two HDGMs constructed by SLAF-seq and CHIP strategies. Bioinformatics and co-expression network analysis revealed that a total of 51 genes were involved in the pathways related to lipid biosynthesis. Further expression analysis confirmed that eight hub genes in the network exhibited dynamic expression at different stages of cotton ovule development and seed maturation via RNA-seq verification. They are all involved in FA biosynthesis, TAG and oil body assembly. These findings not only deepen our understanding of the intricate regulatory processes that control oil accumulation but also provide a foundation for further identification of the causative loci using transcriptome and linkage analyses. These results also provide fundamental genetic resources for future breeding and genetic practices through MAS.

## Figures and Tables

**Table 1:** Statistical analysis of KOC of parents and RILs.

Environment	Parents				RILs							
	LMY 28	XLZ 24	Mean ± SD	SE Mean	Range	Variance	CV(%)	Min	Max	Skewness	Kurtosis	H^2^
14AY	27.59	27.95 **	28.60 ± 1.71	0.11	25.77–35.55	2.91	5.96	25.77	35.55	1.53	4.09	80.07
15AY	30.08	31.03 **	26.86 ± 2.06	0.14	23.47–36.00	4.22	7.65	23.47	36.00	1.91	5.46	83.81
16AY	28.75	29.59 **	27.71 ± 2.03	0.13	24.11–35.27	4.12	7.33	24.11	35.27	1.70	4.17	94.29
14KEL	26.06	27.88 **	29.20 ± 1.93	0.13	25.55–37.13	3.70	6.59	25.55	37.13	1.74	4.11	87.59
15ALE	25.37	27.78 **	27.32 ± 1.74	0.12	24.22–35.70	3.04	6.38	24.22	35.70	1.43	3.01	83.71
BLUE	27.17	28.59 **	28.01 ± 1.73	0.11	25.46–35.36	3.01	6.19	25.46	35.36	2.09	5.75	91.50

Note: LMY28, Lumianyan 28; XLZ24, Xinluzao 24; **, indicates the difference between kernel oil contents of LMY28 and XLZ24 reaches a significant level of *p* < 0.01; SD, standard deviation; SE, standard error; CV, coefficient of variation; Min, minimum value; Max, maximum value; H^2^, broad-sense heritability.

**Table 2:** Brief description of 8 key genes that differentially expressed in ovule stages.

Gene ID	Gene Name	Functional Description	Pathways	Localization	MCODE	References
*GH_A05G3915*	*BCCP2*	Biotin carboxyl carrier protein of heteromeric ACCase	Plastid FA synthesis	Plastid	1	[23,26]
*GH_D03G1283*	*HSD1*	Steroleosin	Oil storage	Oil body	2	
*GH_D03G0050*	*PLDdelta*	Phospholipase D delta	Lipid signaling		2	
*GH_D03G0938*	*LACS4*	lipid biosynthesis and cellular lipid metabolic process	FA elongation	Plastid	3	[7]
*GH_D03G1424*/*GH_D03G1431*	*KCR1*	Ketoacyl CoA reductase	FA elongation	Endomembrane	1	*GH_D03G1424*/*GH_D03G1431*
*GH_D03G1142*	*ENO1*	Phosphoenyl pyruvate enolase	Glycolysis	Plastid	2	*GH_D03G1142*
*GH_D05G0425*	*SQD1*	UDP-Sulfoquinovose synthase	Plastidal glycerolipid, galactolipid and sulfolipid synthesis		3	*GH_D05G0425*
*GH_D09G1772*	*ACX4*	Acyl CoA oxidase	beta-oxidation		1	*GH_D09G1772*

## Data Availability

The datasets generated during and/or analyzed during the current study are available from the corresponding author upon reasonable request.

## Supplementary Materials

The following supporting information can be downloaded at https://www.mdpi.com/article/10.3390/ijms242316595/s1.

## References

[1] Wang W., Sun Y., Yang P., Cai X., Yang L., Ma J., Ou Y., Liu T., Ali I., Liu D. (2019). A high density SLAF-seq SNP genetic map and QTL for seed size, oil and protein content in upland cotton. BMC Genom..

[2] Wendel J.F., Cronn R.C. (2003). Polyploidy and the evolutionary history of cotton. Adv. Agron..

[3] Shang L., Abduweli A., Wang Y., Hua J. (2016). Genetic analysis and QTL mapping of oil content and seed index using two recombinant inbred lines and two backcross populations in Upland cotton. Plant Breed..

[4] Xu Z., Li J., Guo X., Jin S., Zhang X. (2016). Metabolic engineering of cottonseed oil biosynthesis pathway via RNA interference. Sci. Rep..

[5] Zhang Z., Gong J., Zhang Z., Gong W., Li J., Shi Y., Liu A., Ge Q., Pan J., Fan S. (2022). Identification and analysis of oil candidate genes reveals the molecular basis of cottonseed oil accumulation in *Gossypium hirsutum* L. Theor. Appl. Genet..

[6] Dhamodaran G., Krishnan R., Pochareddy Y.K., Pyarelal H.M., Sivasubramanian H., Ganeshram A.K. (2017). A comparative study of combustion, emission, and performance characteristics of rice-bran-, neem-, and cottonseed-oil biodiesels with varying degree of unsaturation. Fuel.

[7] Gong J., Peng Y., Yu J., Pei W., Zhang Z., Fan D., Liu L., Xiao X., Liu R., Lu Q. (2022). Linkage and association analyses reveal that hub genes in energy-flow and lipid biosynthesis pathways form a cluster in upland cotton. Comput. Struct. Biotechnol. J..

[8] Paterson A.H., Wendel J.F., Gundlach H., Guo H., Jenkins J., Jin D., Llewellyn D., Showmaker K.C., Shu S., Udall J. (2012). Repeated polyploidization of *Gossypium* genomes and the evolution of spinnable cotton fibres. Nature.

[9] Hu Y., Chen J., Fang L., Zhang Z., Ma W., Niu Y., Ju L., Deng J., Zhao T., Lian J. (2019). *Gossypium barbadense* and *Gossypium hirsutum* genomes provide insights into the origin and evolution of allotetraploid cotton. Nat. Genet..

[10] Chen Z.J., Sreedasyam A., Ando A., Song Q., De Santiago L.M., Hulse-Kemp A.M., Ding M., Ye W., Kirkbride R.C., Jenkins J. (2020). Genomic diversifications of five *Gossypium* allopolyploid species and their impact on cotton improvement. Nat. Genet..

[11] Ali I., Teng Z., Bai Y., Yang Q., Hao Y., Hou J., Jia Y., Tian L., Liu X., Tan Z. (2018). A high density SLAF-SNP genetic map and QTL detection for fibre quality traits in *Gossypium hirsutum*. BMC Genom..

[12] Yang H., Li C., Lam H.-M., Clements J., Yan G., Zhao S. (2015). Sequencing consolidates molecular markers with plant breeding practice. Theor. Appl. Genet..

[13] Shockey J., Dowd M., Mack B., Gilbert M., Scheffler B., Ballard L., Frelichowski J., Mason C. (2017). Naturally occurring high oleic acid cottonseed oil: Identification and functional analysis of a mutant allele of *Gossypium barbadense* fatty acid desaturase-2. Planta.

[14] Liu Q., Singh S.P., Brubaker C.L., Sharp P.J., Green A.G., Marshall D. (1999). Molecular cloning and expression of a cDNA encoding a microsomal w-6 fatty acid desaturase from cotton (*Gossypium hirsutum*). Funct. Plant Biol..

[15] Sharif I., Farooq J., Chohan S.M., Saleem S., Kainth R.A., Mahmood A., Sarwar G. (2019). Strategies to enhance cottonseed oil contents and reshape fatty acid profile employing different breeding and genetic engineering approaches. J. Integr. Agr..

[16] Chapman K.D., Austin-Brown S., Sparace S.A., Kinney A.J., Ripp K.G., Pirtle I.L., Pirtle R.M. (2001). Transgenic cotton plants with increased seed oleic acid content. J. Am. Oil Chem. Soc..

[17] Hölzl G., Dörmann P. (2019). Chloroplast lipids and their biosynthesis. Annu. Rev. Plant Biol..

[18] Shang X., Cheng C., Ding J., Guo W. (2017). Identification of candidate genes from the SAD gene family in cotton for determination of cottonseed oil composition. Mol. Genet. Genom..

[19] Wang N., Ma J., Pei W., Wu M., Li H., Li X., Yu S., Zhang J., Yu J. (2017). A genome-wide analysis of the lysophosphatidate acyltransferase (LPAAT) gene family in cotton: Organization, expression, sequence variation, and association with seed oil content and fiber quality. BMC Genom..

[20] Zhao Y., Liu Z., Wang X., Wang Y., Hua J. (2018). Molecular Characterization and Expression Analysis of *GhWRI1* in Upland Cotton. J. Plant Biol..

[21] Du X., Liu S., Sun J., Zhang G., Jia Y., Pan Z., Xiang H., He S., Xia Q., Xiao S. (2018). Dissection of complicate genetic architecture and breeding perspective of cottonseed traits by genome-wide association study. BMC Genom..

[22] Zhao Y., Huang Y., Wang Y., Cui Y., Liu Z., Hua J. (2018). RNA interference of *GhPEPC2* enhanced seed oil accumulation and salt tolerance in Upland cotton. Plant Sci..

[23] Zhu D., Le Y., Zhang R., Li X., Lin Z. (2020). A global survey of the gene network and key genes for oil accumulation in cultivated tetraploid cottons. Plant Biotechnol. J..

[24] Liu R., Gong J., Xiao X., Zhang Z., Li J., Liu A., Lu Q., Shang H., Shi Y., Ge Q. (2018). GWAS Analysis and QTL Identification of Fiber Quality Traits and Yield Components in Upland Cotton Using Enriched High-Density SNP Markers. Front. Plant Sci..

[25] Li-Beisson Y., Shorrosh B., Beisson F., Andersson M.X., Arondel V., Bates P.D., Baud S., Bird D., Debono A., Durrett T.P. (2013). Acyl-lipid metabolism. Arab. Book.

[26] Turquetti-Moraes D.K., Moharana K.C., Almeida-Silva F., Pedrosa-Silva F., Venancio T.M. (2022). Integrating omics approaches to discover and prioritize candidate genes involved in oil biosynthesis in soybean. Gene..

[27] Banda T., Kumarasamy M. (2020). Application of Multivariate Statistical Analysis in the Development of a Surrogate Water Quality Index (WQI) for South African Watersheds. Water.

[28] Rehman F., Saeed A., Yaseen M., Shakeel A., Ziaf K., Munir H., Tariq S.A., Raza M.A., Riaz A. (2019). Genetic evaluation and characterization using cluster heat map to assess NaCl tolerance in tomato germplasm at the seedling stage. Chil. J. Agr. Res..

[29] Liu X., Teng Z., Wang J., Wu T., Zhang Z., Deng X., Fang X., Tan Z., Ali I., Liu D. (2018). Enriching an intraspecific genetic map and identifying QTL for fiber quality and yield component traits across multiple environments in Upland cotton (*Gossypium hirsutum* L.). Mol. Genet. Genom..

[30] Zhao Y., Wang Y., Huang Y., Cui Y., Hua J. (2018). Gene network of oil accumulation reveals expression profiles in developing embryos and fatty acid composition in Upland cotton. J. Plant Physiol..

[31] Zhao W., Kong X., Yang Y., Nie X., Lin Z. (2019). Association mapping seed kernel oil content in upland cotton using genome-wide SSRs and SNPs. Mol. Breed..

[32] Zhu D., Li X., Wang Z., You C., Nie X., Sun J., Zhang X., Zhang D., Lin Z. (2020). Genetic dissection of an allotetraploid interspecific CSSLs guides interspecific genetics and breeding in cotton. BMC Genom..

[33] Huang A.H.C. (1994). Structure of plant seed oil bodies. Curr. Opin. Struct. Biol..

[34] Kim H.U., Hsieh K., Ratnayake C., Huang A.H. (2002). A novel group of oleosins is present inside the pollen of *Arabidopsis*. J. Biol. Chem..

[35] Huang A.H.C. (2018). Plant Lipid Droplets and Their Associated Proteins: Potential for Rapid Advances. Plant Physiol..

[36] Walther T.C., Chung J., Farese R.V. (2017). Lipid Droplet Biogenesis. Ann. Rev. Cell Dev. Biol..

[37] Baud S., Lepiniec L. (2009). Regulation of de novo fatty acid synthesis in maturing oilseeds of *Arabidopsis*. Plant Physiol. Biochem..

[38] Cai G., Fan C., Liu S., Yang Q., Liu D., Wu J., Li J., Zhou Y., Guo L., Wang X. (2020). Nonspecific phospholipase C6 increases seed oil production in oilseed *Brassicaceae* plants. N. Phytol..

[39] Fatland B.L., Ke J., Anderson M.D., Mentzen W.I., Cui L.W., Allred C.C., Johnston J.L., Nikolau B.J., Wurtele E.S. (2002). Molecular characterization of a heteromeric ATP-citrate lyase that generates cytosolic acetyl-coenzyme A in *Arabidopsis*. Plant Physiol..

[40] Fatland B.L., Nikolau B.J., Wurtele E.S. (2005). Reverse genetic characterization of cytosolic acetyl-CoA generation by ATP-citrate lyase in *Arabidopsis*. Plant Cell.

[41] Yin Y., Guo Z., Chen K., Tian T., Tan J., Chen X., Chen J., Yang B., Tang S., Peng K. (2020). Ultra-High α-Linolenic Acid Accumulating Developmental Defective Embryo was Rescued by Lysophosphatidic Acid Acyltransferase 2. Plant J..

[42] Prabhakar V., Lottgert T., Gigolashvili T., Bell K., Flugge U.I., Hausler R.E. (2009). Molecular and functional characterization of the plastid-localized Phosphoenolpyruvate enolase (ENO1) from *Arabidopsis thaliana*. FEBS Lett..

[43] Bao B., Chao H., Wang H., Zhao W., Zhang L., Raboanatahiry N., Wang X., Wang B., Jia H., Li M. (2018). Stable, Environmental Specific and Novel QTL Identification as Well as Genetic Dissection of Fatty Acid Metabolism in *Brassica napus*. Front. Plant Sci..

[44] Contento A.L., Bassham D.C. (2010). Increase in catalase-3 activity as a response to use of alternative catabolic substrates during sucrose starvation. Plant Physiol. Biochem..

[45] Zhong Y., Wang Y., Li P., Gong W., Wang X., Yan H., Ge Q., Liu A., Shi Y., Shang H. (2023). Genome-Wide Analysis and Functional Characterization of LACS Gene Family Associated with Lipid Synthesis in Cotton (*Gossypium* spp.). Int. J. Mol. Sci..

[46] Jessen D., Olbrich A., Knüfer J., Krüger A., Hoppert M., Polle A., Fulda M. (2011). Combined activity of LACS1 and LACS4 is required for proper pollen coat formation in *Arabidopsis*. Plant J..

[47] Shimojima M. (2011). Biosynthesis and functions of the plant sulfolipid. Prog. Lipid Res..

[48] Baud S., Dichow N.R., Kelemen Z., d’Andréa S., To A., Berger N., Canonge M., Kronenberger J., Viterbo D., Dubreucq B. (2009). Regulation of HSD1 in seeds of *Arabidopsis thaliana*. Plant Cell Physiol..

[49] Khan B.R., Adham A.R., Zolman B.K. (2012). Peroxisomal Acyl-CoA oxidase 4 activity differs between *Arabidopsis* accessions. Plant Mol. Biol..

[50] Yang Z., Liu Z., Ge X., Lu L., Qin W., Qanmber G., Liu L., Wang Z., Li F. (2023). Brassinosteroids regulate cotton fiber elongation by modulating very-long-chain fatty acid biosynthesis. Plant Cell.

[51] Qu C., Jia L., Fu F., Zhao H., Lu K., Wei L., Xu X., Liang Y., Li S., Wang R. (2017). Genome-wide association mapping and Identification of candidate genes for fatty acid composition in *Brassica napus* L. using SNP markers. BMC Genom..

[52] Kogure K., Watanabe A., Ito Y. (2022). Interaction of ONION2 ketoacyl CoA synthase with ketoacyl CoA reductase of rice. Mol. Biol. Rep..

[53] Gu J., Chao H., Wang H., Li Y., Li D., Xiang J., Gan J., Lu G., Zhang X., Long Y. (2017). Identification of the relationship between oil body morphology and oil content by microstructure comparison combining with QTL analysis in *Brassica napus*. Front. Plant Sci..

[54] Mulichak A.M., Theisen M.J., Essigmann B., Benning C., Garavito R.M. (1999). Crystal structure of SQD1, an enzyme involved in the biosynthesis of the plant sulfolipid headgroup donor UDP-sulfoquinovose. Proc. Natl. Acad. Sci. USA.

[55] Nakajima Y., Umena Y., Nagao R., Endo K., Kobayashi K., Akita F., Suga M., Wada H., Noguchi T., Shen J.R. (2018). Thylakoid membrane lipid sulfoquinovosyl-diacylglycerol (SQDG) is required for full functioning of photosystem II in *Thermosynechococcus elongatus*. J. Biol. Chem..

[56] Nguyen V.C., Nakamura Y. (2023). Distinctly localized lipid phosphate phosphatases mediate endoplasmic reticulum glycerolipid metabolism in *Arabidopsis*. Plant Cell.

[57] Degenkolbe T., Giavalisco P., Zuther E., Seiwert B., Hincha D.K., Willmitzer L. (2012). Differential remodeling of the lipidome during cold acclimation in natural accessions of *Arabidopsis thaliana*. Plant J..

[58] Hafeez A., Ge Q., Zhang Q., Li J., Gong J., Liu R., Shi Y., Shang H., Liu A., Iqbal M.S. (2021). Multi-responses of O-methyltransferase genes to salt stress and fiber development of *Gossypium* species. BMC Plant Biol..

[59] Verslues P.E., Agarwal M., Katiyar-Agarwal S., Zhu J., Zhu J.-K. (2006). Methods and concepts in quantifying resistance to drought, salt and freezing, abiotic stresses that affect plant water status. Plant J..

[60] Krawczyk H.E., Rotsch A.H., Herrfurth C., Scholz P., Shomroni O., Salinas-Riester G., Feussner I., Ischebeck T. (2022). Heat stress leads to rapid lipid remodeling and transcriptional adaptations in *Nicotiana tabacum* pollen tubes. Plant Physiol..

[61] Li F., Asami T., Wu X., Tsang E.W.T., Cutler A.J. (2007). A Putative Hydroxysteroid Dehydrogenase Involved in Regulating Plant Growth and Development. Plant Physiol..

[62] Zhang Z., Cheng Z.-J., Gan L., Zhang H., Wu F.-Q., Lin Q.-B., Wang J.-L., Wang J., Guo X.-P., Zhang X. (2016). OsHSD1, a hydroxysteroid dehydrogenase, is involved in cuticle formation and lipid homeostasis in rice. Plant Sci..

[63] Shao Q., Liu X., Su T., Ma C., Wang P. (2019). New Insights into the Role of Seed Oil Body Proteins in Metabolism and Plant Development. Front. Plant Sci..

[64] Kunst L., Samuels L. (2009). Plant cuticles shine: Advances in wax biosynthesis and export. Curr. Opin. Plant Biol..

[65] Voelker T., Kinney A.J. (2001). Variation in the Biosynthesis of Seed-Storage Lipids. Annu. Rev. Plant Physiol. Plant Mol. Biol..

[66] Chao H., Wang H., Wang X., Guo L., Gu J., Zhao W., Li B., Chen D., Raboanatahiry N., Li M. (2017). Genetic dissection of seed oil and protein content and identification of networks associated with oil content in *Brassica napus*. Sci. Rep..

[67] Wang S., Basten C.J., Zeng Z. (2012). Windows QTL Cartographer.

[68] Zeng Z.-B. (1994). Precision mapping of quantitative trait loci. Genetics.

[69] Sun F.-D., Zhang J.-H., Wang S.-F., Gong W.-K., Shi Y.-Z., Liu A.-Y., Li J.-W., Gong J.-W., Shang H.-H., Yuan Y.-L. (2012). QTL mapping for fiber quality traits across multiple generations and environments in upland cotton. Mol. Breed..

