# Supplementary material for: Hub Genes in Stable QTLs Orchestrate the Accumulation of Cottonseed Oil in Upland Cotton via Catalyzing Key Steps of Lipid-Related Pathways
Source: Int J Mol Sci. 2023 Nov 22;24(23):16595. doi: 10.3390/ijms242316595 (PMC10706765; doi:10.3390/ijms242316595)
Supplement: Supplementary file 1 [file ijms-24-16595-s001.zip › Suplementary figures.pdf]

Supplementary Figures

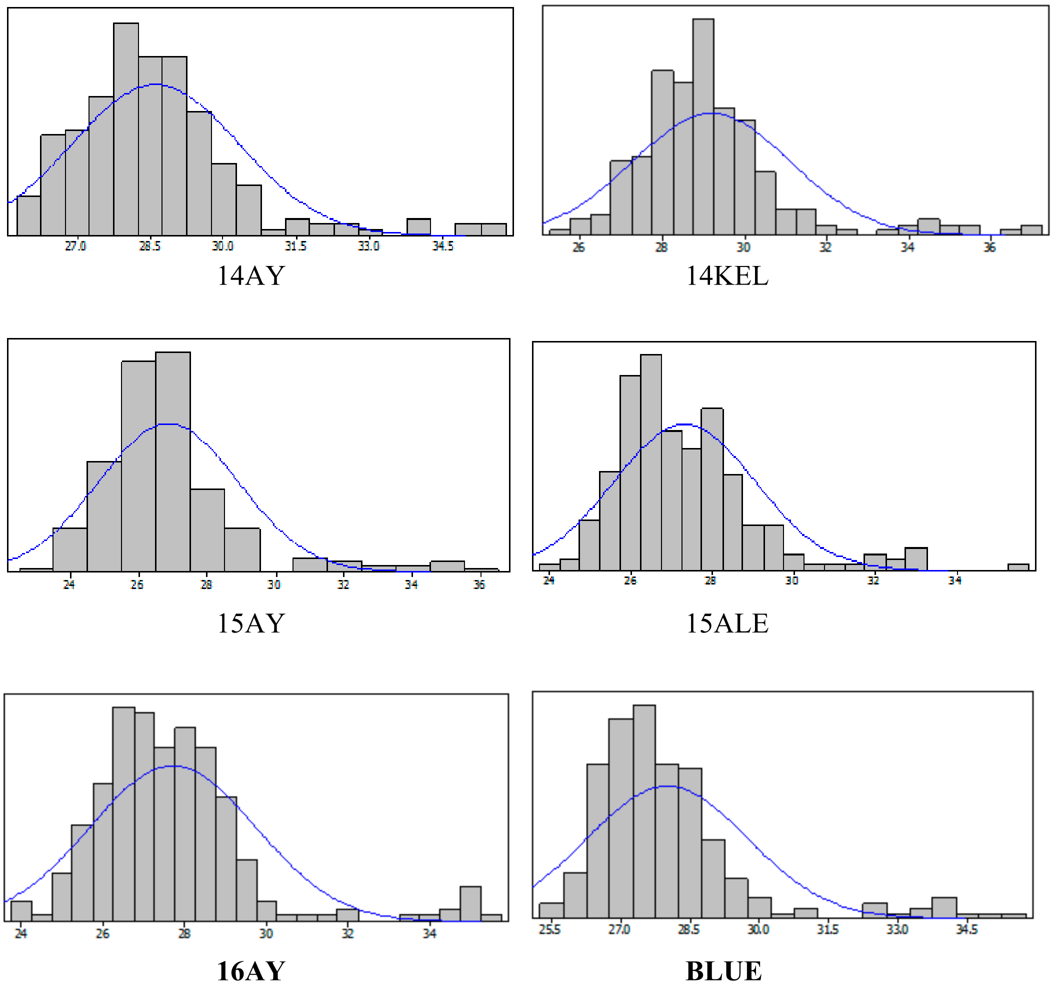

Fig. S1 Frequency distribution histograms of KOC of the RIL population in each environment

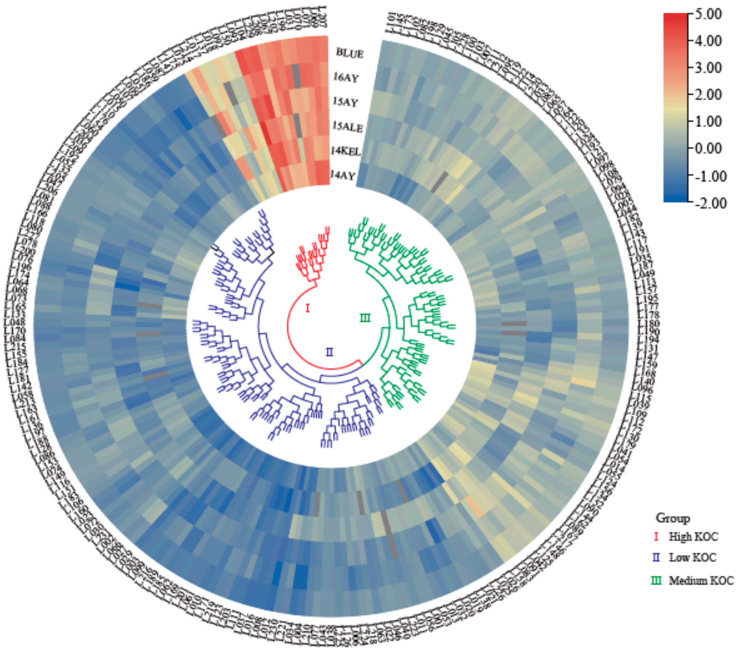

Fig. S2 Hierarchical clustering heatmap of the RIL population across multiple environments at optimal clustering number (k=3)

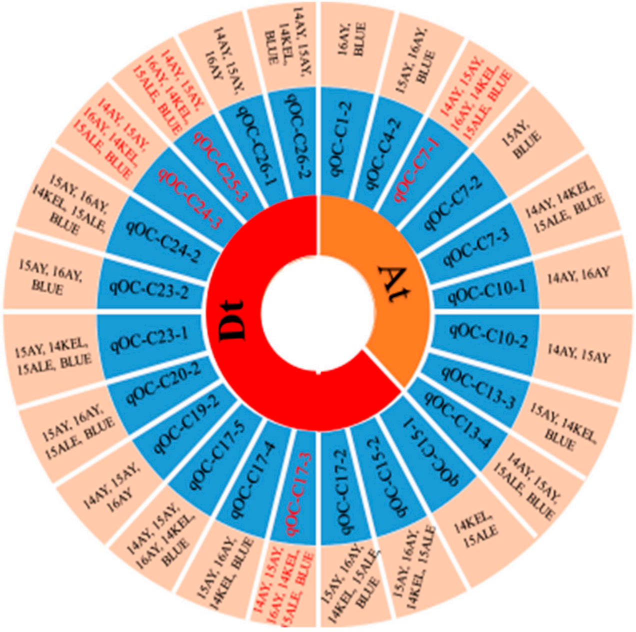

Fig. S3 Summary of distribution of stable QTLs identified via SLAF-seq strategy on At and Dt sub-genomes

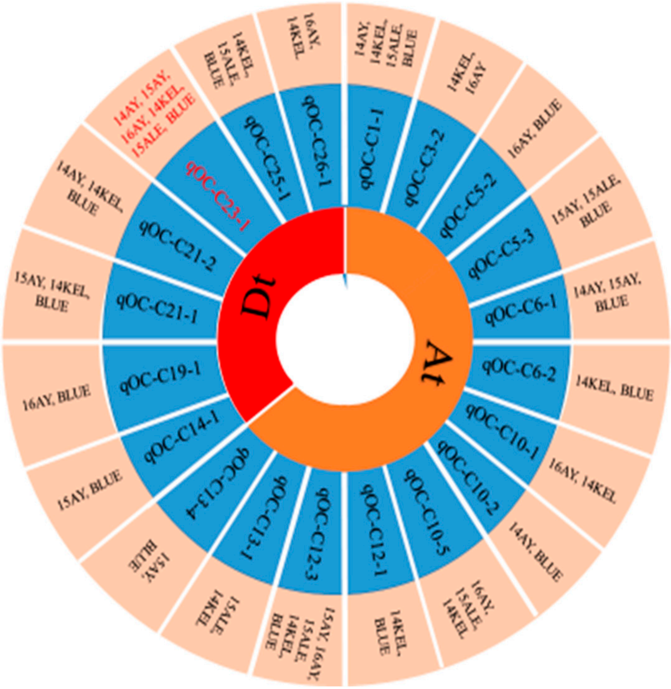

Fig. S4 Summary of distribution of stable QTLs identified via CHIP strategy on At and Dt sub-genomes

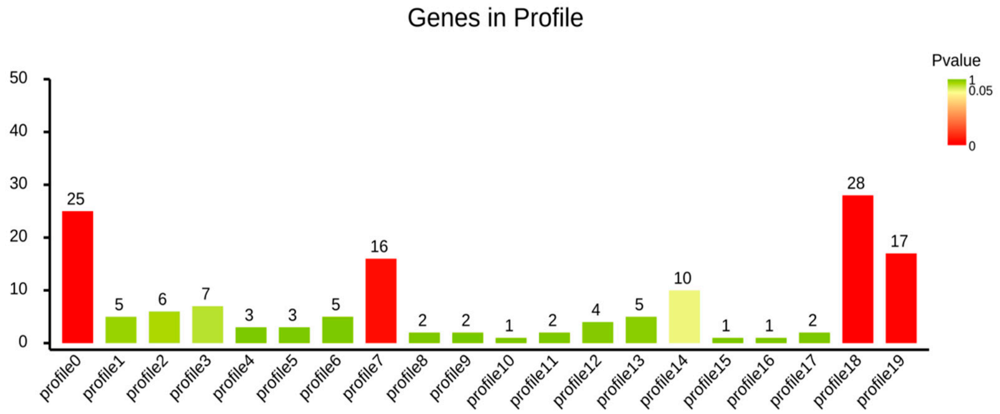

Fig. S5 Dynamic expression pattern organizes QTL candidate genes into 19 cluster profiles
