# Supplementary material for: Hub Genes in Stable QTLs Orchestrate the Accumulation of Cottonseed Oil in Upland Cotton via Catalyzing Key Steps of Lipid-Related Pathways
Source: Int J Mol Sci. 2023 Nov 22;24(23):16595. doi: 10.3390/ijms242316595 (PMC10706765; doi:10.3390/ijms242316595)
Supplement: Supplementary file 1 [file ijms-24-16595-s001.zip › Table S1.pdf]

Table S1 Brief depiction of the HDGMs of the RIL population

Chromosome ID	SLAF-seq. genetic map			CHIP genetic map		
	No. markers	Length (cM)	Average marker interval (cM)	No. of markers	Length (cM)	Average marker interval (cM)
Chr01	81	125.45	1.55	236	112.38	0.49
Chr02	68	170.63	2.51	61	57.22	0.97
Chr03	205	144.86	0.71	310	85.26	0.28
Chr04	94	179.64	1.91	226	98.49	0.44
Chr05	166	153.29	0.92	294	163.46	0.57
Chr06	99	169.47	1.71	187	85.28	0.46
Chr07	608	174.41	0.29	231	145.52	0.66
Chr08	306	128.84	0.42	481	142.55	0.32
Chr09	253	127.37	0.50	225	107.03	0.49
Chr10	101	173.24	1.72	103	78.66	0.78
Chr11	105	173.16	1.65	205	109.33	0.57
Chr12	394	148.73	0.38	397	157.24	0.41
Chr13	821	150.18	0.18	344	132.40	0.40
A_t sub-genome	3301	2019.26	1.11	3300	1474.63	0.45
Chr14	116	163.18	1.41	319	112.26	0.36
Chr15	25	129.43	5.18	124	69.36	0.64
Chr16	149	141.82	0.95	55	53.91	0.98
Chr17	17	172.91	10.17	19	60.60	3.56
Chr18	68	172.08	2.53	58	45.05	0.79
Chr19	91	153.36	1.69	240	103.53	0.44
Chr20	185	165.77	0.90	145	84.74	0.59
Chr21	82	140.43	1.71	150	127.93	0.87
Chr22	25	42.97	1.72	93	83.17	0.92
Chr23	48	130.45	2.72	94	57.14	0.63
Chr24	30	130.27	4.34	113	57.89	0.52
Chr25	38	127.49	3.36	82	87.29	1.06
Chr26	191	137.48	0.72	59	60.49	1.06
D_t sub-genome	1065	1807.65	2.87	1551	1003.36	0.65
Total	4366	3826.92	1.99	4851	2477.99	0.51
