# Supplementary material for: Hub Genes in Stable QTLs Orchestrate the Accumulation of Cottonseed Oil in Upland Cotton via Catalyzing Key Steps of Lipid-Related Pathways
Source: Int J Mol Sci. 2023 Nov 22;24(23):16595. doi: 10.3390/ijms242316595 (PMC10706765; doi:10.3390/ijms242316595)
Supplement: Supplementary file 1 [file ijms-24-16595-s001.zip › Table S2.pdf]

Table S2 Analysis of variance of KOC trait under multiple environments

Env.	Source	Df	Sum sq	Mean sq	F-value	Pr(>F)
14AY	Replication	1	16.44	16.4409	11.0110	0.001054 **
	Genotype	230	1437.39	6.2495	4.1855	< 2.2e-16 ***
	Residuals	228	340.43	1.4931		
15AY	Replication	1	50.52	50.519	35.1416	1.125e-08 ***
	Genotype	230	1872.06	8.1390	5.6619	< 2.2e-16 ***
	Residuals	228	327.77	1.4380		
16AY	Replication	1	3.12	3.1157	3.5250	0.06172
	Genotype	230	1915.24	8.3271	9.4211	< 2e-16 ***
	Residuals	230	203.29	0.8839		
14KEL	Replication	1	20.80	20.8006	7.9510	0.005226 **
	Genotype	230	2121.46	9.2238	3.5257	< 2.2e-16 ***
	Residuals	230	601.71	2.6161		
15ALE	Replication	1	24.53	24.5342	22.1283	4.4e-06 ***
	Genotype	230	1431.46	6.2237	5.6134	< 2e-16 ***
	Residuals	230	255.01	1.1087		
BLUE	Replication	1	0.0	0.0000	2.4160e-01	0.6236
	Genotype	230	1328.3	5.7753	6.5953e+27	< 2e-16 ***
	Residuals	230	0.0	0.0000		

Significant codes: '***' 0.001, '**' 0.01, '*' 0.05.
