# Supplementary material for: Hub Genes in Stable QTLs Orchestrate the Accumulation of Cottonseed Oil in Upland Cotton via Catalyzing Key Steps of Lipid-Related Pathways
Source: Int J Mol Sci. 2023 Nov 22;24(23):16595. doi: 10.3390/ijms242316595 (PMC10706765; doi:10.3390/ijms242316595)
Supplement: Supplementary file 1 [file ijms-24-16595-s001.zip › Table S3.pdf]

Table S3 Statistical analysis of the three groups of high, medium and low KOC in each environment

Env.	grouping	N	Mean value	Standard deviation	Varian ce	Min. value	Max. value	Skewness	Kurtosis	CV
14AY	High KOC	19	32.62	1.94	3.76	29.57	35.55	0.07	-1.20	5.94
	Low KOC	123	27.74	0.95	0.90	25.77	29.82	-0.03	-0.85	3.42
	Medium KOC	89	28.93	0.97	0.95	26.64	30.92	-0.20	-0.51	3.36
14KEL	High KOC	19	33.81	2.21	4.88	29.55	37.13	-0.42	-0.64	6.53
	Low KOC	117	28.08	0.90	0.81	25.55	29.72	-0.41	-0.28	3.20
	Medium KOC	86	29.71	0.87	0.75	28.18	31.87	0.53	-0.15	2.91
15ALE	High KOC	18	31.66	1.70	2.90	28.68	35.70	0.36	0.46	5.38
	Low KOC	122	26.45	0.97	0.94	24.22	29.70	0.46	0.35	3.67
	Medium KOC	89	27.63	1.01	1.02	25.36	29.58	-0.12	-0.75	3.65
15AY	High KOC	18	32.18	2.37	5.62	27.55	36.00	-0.31	-0.65	7.36
	Low KOC	122	25.80	0.99	0.97	23.47	28.26	0.10	-0.32	3.82
	Medium KOC	89	27.24	1.02	1.03	24.96	30.88	0.60	1.00	3.73
16AY	High KOC	17	33.03	2.25	5.06	29.15	35.27	-0.60	-1.26	6.81
	Low KOC	123	26.48	0.88	0.78	24.11	28.60	-0.24	0.23	3.34
	Medium KOC	89	28.39	0.80	0.64	26.02	30.95	0.09	0.92	2.82
BLUE	High KOC	19	32.73	1.76	3.11	29.99	35.36	-0.33	-1.15	5.39
	Low KOC	123	26.95	0.52	0.27	25.46	27.80	-0.54	-0.29	1.93
	Medium KOC	89	28.45	0.53	0.28	27.36	29.80	0.41	-0.24	1.85
