# Supplementary material for: Hub Genes in Stable QTLs Orchestrate the Accumulation of Cottonseed Oil in Upland Cotton via Catalyzing Key Steps of Lipid-Related Pathways
Source: Int J Mol Sci. 2023 Nov 22;24(23):16595. doi: 10.3390/ijms242316595 (PMC10706765; doi:10.3390/ijms242316595)
Supplement: Supplementary file 1 [file ijms-24-16595-s001.zip › Table S4.pdf]

Table S4 KOC performance of the RILs across different environments and their phenotypic clustering result

Lines	14AY	14KEL	15ALE	15AY	16AY	BLUE	Phenotypic clustering
L017	29.57	30.19	29.63	31.46	30.3	30.19	High KOC
L018	31.38	32.72	31.87	27.55	NA	31.16	High KOC
L031	33.91	35.25	31.31	31.81	NA	33.23	High KOC
L067	32.46	34.31	31.11	33.26	35.02	33.39	High KOC
L069	35.24	33.76	33.01	34.05	34.63	34.14	High KOC
L070	34.04	34.96	32.03	31.34	35.24	33.76	High KOC
L071	35.47	33.49	31.92	32.4	35.08	33.77	High KOC
L075	30.05	34.42	29.77	NA	29.96	31.14	High KOC
L092	31.26	31.22	30.52	29.37	29.42	30.36	High KOC
L093	35.55	36.65	35.70	35.17	33.92	35.36	High KOC
L104	33.23	36.76	33.08	34.74	35.06	34.70	High KOC
L137	30.50	29.55	28.68	29.4	31.48	30.01	High KOC
L150	30.03	31.71	29.96	28.73	29.15	29.99	High KOC
L199	32.20	31.15	NA	31.56	35.27	32.74	High KOC
L202	32.39	35.61	32.48	34.61	34.48	34.00	High KOC
L207	34.94	34.09	32.89	33.14	33.27	33.66	High KOC
L208	32.15	35.08	32.51	31.08	31.99	32.7	High KOC
L214	31.36	34.36	30.14	33.57	32.11	32.33	High KOC
L229	34.04	37.13	33.22	36.00	35.04	35.14	High KOC
L005	29.37	29.02	28.48	27.03	27.18	28.22	Medium KOC
L007	27.58	30.29	28.78	27.23	27.98	28.49	Medium KOC
L014	30.01	28.62	28.82	26.53	26.02	27.94	Medium KOC
L024	27.83	28.80	27.45	27.03	28.65	28.07	Medium KOC
L028	29.59	29.30	27.81	27.86	26.73	28.20	Medium KOC
L033	28.72	30.74	28.5	27.49	29.13	29.06	Medium KOC
L035	29.67	28.68	27.4	27.09	28.29	28.27	Medium KOC
L039	30.92	28.88	28.65	28.83	28.10	28.99	Medium KOC
L041	29.82	30.53	27.96	26.39	28.03	28.66	Medium KOC
L042	28.95	30.14	29.27	29.16	29.21	29.38	Medium KOC
L044	29.61	29.1	28.40	27.15	27.57	28.38	Medium KOC
L045	28.06	29.83	27.08	26.34	27.74	27.94	Medium KOC
L049	29.52	28.26	26.71	25.93	28.16	27.81	Medium KOC
L054	30.26	30.40	27.76	25.99	27.48	28.47	Medium KOC
L055	28.94	28.79	28.61	26.87	27.49	28.17	Medium KOC
L057	27.88	29.92	27.85	26.04	28.87	28.32	Medium KOC
L065	28.51	30.06	27.34	26.93	29.24	28.59	Medium KOC
L079	29.07	29.90	27.83	27.06	27.81	28.40	Medium KOC
L082	28.47	30.07	27.27	27.23	27.94	28.29	Medium KOC
L083	29.74	31.42	28.49	30.88	28.73	29.80	Medium KOC
L085	29.24	31.71	28.83	27.26	28.20	29.17	Medium KOC

L089	30.35	30.02	26.62	27.27	28.02	28.50	Medium KOC
L090	29.61	29.80	26.6	27.72	28.04	28.39	Medium KOC
L091	29.28	30.36	29.34	29.14	28.22	29.25	Medium KOC
L094	29.38	29.47	28.14	26.98	27.83	28.41	Medium KOC
L095	28.58	30.38	28.77	27.02	27.49	28.52	Medium KOC
L096	29.33	28.18	27.11	28.63	29.74	28.64	Medium KOC
L097	29.54	29.83	28.14	27.46	28.51	28.77	Medium KOC
L098	29.75	29.73	27.63	27.05	28.99	28.74	Medium KOC
L100	27.51	28.95	28.16	26.91	27.8	27.95	Medium KOC
L101	27.43	29.09	27.3	28.03	27.42	27.87	Medium KOC
L102	28.25	29.52	27.14	27.47	27.42	28.00	Medium KOC
L103	26.88	29.46	27.98	27.08	27.69	27.92	Medium KOC
L105	28.19	29.73	27.9	26.68	27.79	28.17	Medium KOC
L108	29.19	29.64	28.61	28.9	27.72	28.77	Medium KOC
L109	30.7	30.18	28.13	28.72	28.84	29.32	Medium KOC
L112	30.47	28.46	28.85	26.52	28.57	28.63	Medium KOC
L113	29.91	28.65	26.75	26.35	29.36	28.34	Medium KOC
L114	29.43	30.80	29.49	27.71	27.94	29.13	Medium KOC
L115	27.67	28.95	27.86	28.14	30.95	28.90	Medium KOC
L117	29.82	29.68	26.7	26.57	28	28.24	Medium KOC
L119	28.8	30.70	28.53	27.10	28.48	28.85	Medium KOC
L120	29.12	29.07	28.17	26.64	29.21	28.58	Medium KOC
L121	29.61	29.70	28.27	28.23	29.06	29.03	Medium KOC
L122	29.09	29.05	27.63	26.49	29.07	28.4	Medium KOC
L124	28.59	28.94	28.91	26.55	26.96	28.02	Medium KOC
L125	29.57	30.66	28.79	28.86	29.44	29.53	Medium KOC
L129	28.1	29.81	27.94	26.16	26.79	27.84	Medium KOC
L130	30.64	29.82	29.55	27.29	29.59	29.47	Medium KOC
L131	28.26	28.80	25.36	26.96	28.24	27.62	Medium KOC
L134	30.4	31.30	26.21	28.39	27.77	28.83	Medium KOC
L135	28.89	28.90	28.26	27.08	28.91	28.51	Medium KOC
L139	29.73	28.92	25.75	27.36	29.01	28.24	Medium KOC
L140	26.84	30.25	26.19	28.2	29.61	28.40	Medium KOC
L143	29.05	28.74	26.07	27.18	28.56	28.00	Medium KOC
L144	29.38	30.21	26.49	28.31	28.35	28.59	Medium KOC
L145	27.4	29.95	27.39	27.93	26.96	27.95	Medium KOC
L146	28.31	31.87	27.71	28.53	28.30	29.04	Medium KOC
L147	27.8	28.62	26.43	27.66	28.06	27.77	Medium KOC
L148	28.47	31.69	27.79	28.4	28.4	29.05	Medium KOC
L152	29.19	29.46	26.33	28.41	28.76	28.47	Medium KOC
L156	29.24	30.97	26.60	26.58	29.81	28.87	Medium KOC
L157	28.93	29.81	26.28	26.19	27.78	27.92	Medium KOC
L158	30.24	31.87	27.90	28.80	29.27	29.70	Medium KOC
L159	27.91	29.03	26.33	26.95	28.67	27.91	Medium KOC

L162	30.48	30.16	26.13	27.79	29.31	28.87	Medium KOC
L164	28.85	29.91	27.80	25.90	29.06	28.50	Medium KOC
L167	29.84	30.14	26.73	26.68	28.83	28.58	Medium KOC
L168	27.23	28.69	26.12	26.66	28.9	27.69	Medium KOC
L172	30.33	28.98	28.65	27.63	28.24	28.77	Medium KOC
L173	28.49	28.99	27.37	28.17	28.23	28.27	Medium KOC
L177	28.63	28.54	27.46	25.91	27.70	27.74	Medium KOC
L178	28.01	28.99	27.29	25.67	28.72	27.92	Medium KOC
L179	30.2	30.02	29.25	27.65	28.28	29.11	Medium KOC
L180	28.19	NA	27.08	25.09	28.35	27.36	Medium KOC
L182	28.79	29.72	26.18	26.98	29.23	28.34	Medium KOC
L187	30.13	28.81	26.73	27.17	27.87	28.16	Medium KOC
L190	28.38	29.09	27.18	24.96	28.35	27.79	Medium KOC
L191	29.55	29.23	26.53	26.19	28.71	28.18	Medium KOC
L193	28.14	28.54	28.38	27.03	28.5	28.2	Medium KOC
L194	27.85	NA	25.57	25.69	29.38	27.36	Medium KOC
L195	28.13	29.94	26.17	25.94	28.31	27.88	Medium KOC
L198	28.24	29.92	26.93	27.42	28.37	28.28	Medium KOC
L211	27.11	30.50	27.46	26.97	28.17	28.20	Medium KOC
L212	28.64	30.79	28.21	26.22	28.02	28.54	Medium KOC
L217	28.85	30.51	27.59	29.42	30.23	29.42	Medium KOC
L220	29.02	30.64	29.58	27.36	29.00	29.25	Medium KOC
L222	28.72	NA	28.91	26.89	28.6	28.37	Medium KOC
L228	26.64	29.52	27.91	26.30	28.39	27.94	Medium KOC
L001	28.22	28.01	27.77	25.97	27.09	27.47	Low KOC
L002	26.78	28.53	28.00	25.95	27.63	27.51	Low KOC
L003	27	27.89	25.97	24.75	25.93	26.40	Low KOC
L004	26.49	28.06	27.23	24.19	24.22	26.08	Low KOC
L006	27.48	28.82	27.04	26.51	25.16	26.97	Low KOC
L008	27.49	25.55	25.57	24.46	25.43	25.7	Low KOC
L009	29.29	28.77	26.12	25.73	25.99	27.2	Low KOC
L010	26.07	26.99	25.65	25.8	26.12	26.17	Low KOC
L011	27.78	27.76	27.90	25.64	26.69	27.21	Low KOC
L012	26.61	27.03	25.34	24.13	24.11	25.46	Low KOC
L013	29	NA	27.80	25.61	25.39	26.86	Low KOC
L015	28.18	27.44	26.67	25.38	25.41	26.6	Low KOC
L016	27.1	26.34	25.86	24.44	25.21	25.81	Low KOC
L019	28.78	28.35	28.09	24.63	26.77	27.42	Low KOC
L020	28.88	27.87	28.37	23.47	25.71	26.94	Low KOC
L021	28.93	26.82	26.82	27.02	26.92	27.24	Low KOC
L022	29.1	29.52	27.24	25.52	26.26	27.59	Low KOC
L023	26.43	28.54	27.00	25.35	25.54	26.64	Low KOC
L025	28.77	27.24	27.00	27.59	28.29	27.78	Low KOC
L026	28.03	26.25	29.70	26.44	27.53	27.58	Low KOC

L027	26.03	28.85	27.15	25.11	28.60	27.41	Low KOC
L029	26.88	28.05	26.21	24.05	25.24	26.18	Low KOC
L030	26.34	28.22	26.92	25.57	26.59	26.83	Low KOC
L032	28.44	27.24	27.85	26.68	26.07	27.19	Low KOC
L034	26.34	26.86	26.98	24.51	24.31	25.80	Low KOC
L036	27.88	26.98	26.91	25.43	26.81	26.85	Low KOC
L037	26.69	26	25.41	24.42	25.67	25.69	Low KOC
L038	28.52	28.31	27.05	25.91	26.63	27.32	Low KOC
L040	28.28	28.81	28.73	25.33	26.42	27.58	Low KOC
L043	27.93	28.66	27	25.97	26.76	27.33	Low KOC
L046	28.39	28.32	28.68	26.32	26.81	27.72	Low KOC
L047	29.04	28.99	26.32	25.77	27.09	27.52	Low KOC
L048	27.37	27.37	26.11	26.19	26.54	26.74	Low KOC
L050	28.13	27.52	26.95	24.34	25.53	26.54	Low KOC
L051	29.03	28.58	25.63	25.72	25.72	26.94	Low KOC
L052	28.13	28.36	26.24	26.19	27.22	27.3	Low KOC
L053	28.67	27.82	27.96	25.76	25.77	27.17	Low KOC
L056	28.55	27.88	28.01	26.18	26.87	27.52	Low KOC
L058	27.79	29.1	25.28	26.96	26.34	27.11	Low KOC
L059	27.8	28.22	26.27	25.56	24.17	26.35	Low KOC
L060	27.87	27.69	26.04	24.85	26.42	26.66	Low KOC
L061	26.83	27.45	25.71	25.59	26.15	26.4	Low KOC
L062	27.17	28.75	25.13	25.42	25.17	26.37	Low KOC
L063	29.35	NA	27.31	25.27	26	26.95	Low KOC
L064	28.93	28.71	26.01	27.24	26.5	27.45	Low KOC
L066	26.39	27.55	26.56	25.69	28.33	27.07	Low KOC
L068	29.47	NA	26.07	27.33	26.48	27.22	Low KOC
L072	26.2	29.11	27.22	25.57	26.9	27.15	Low KOC
L073	28.72	28.13	25.21	27.47	26.36	27.12	Low KOC
L074	28.32	29.61	26.1	27.46	26.8	27.67	Low KOC
L076	28.2	27.27	24.91	27.09	25.56	26.52	Low KOC
L077	28.17	29.47	27.5	25.3	26.2	27.42	Low KOC
L078	28.02	29.35	25.79	24.45	26.51	26.98	Low KOC
L080	28.69	27.49	26.47	24.91	28.26	27.31	Low KOC
L081	29.75	28.9	26.38	25.65	26.72	27.52	Low KOC
L084	27.28	NA	26.58	25.7	26.26	26.46	Low KOC
L086	27.28	29.31	26.83	27.56	26.37	27.47	Low KOC
L087	27.74	27.91	25.39	25.08	25.28	26.3	Low KOC
L088	29.33	29.52	25.75	25.64	27.27	27.61	Low KOC
L099	25.98	28.67	26.26	24.31	26.46	26.52	Low KOC
L106	26.21	29.11	26.33	27.87	26.86	27.31	Low KOC
L107	29.02	27.46	25.98	25.98	25.2	26.65	Low KOC
L110	28.29	26.78	26.45	23.95	25.19	26.15	Low KOC
L111	26.35	26.22	25.48	25.33	27.02	26.17	Low KOC

L116	27.65	29.4	26.7	26.65	27.96	27.8	Low KOC
L118	28.88	27.94	26.29	24.98	27.3	27.18	Low KOC
L123	26.73	27.66	26.56	25.44	26.67	26.7	Low KOC
L126	27.42	29.08	27.6	25.54	26.25	27.26	Low KOC
L127	27.22	28.19	25.96	25.9	27.23	27	Low KOC
L128	27.58	28.98	26.31	26.77	26.58	27.28	Low KOC
L132	28.58	28.92	26.01	26.73	27.36	27.58	Low KOC
L133	27.3	27.93	26.66	25.91	26.6	26.94	Low KOC
L136	26.59	27.48	26.1	26.94	27.11	26.88	Low KOC
L138	28.56	27.17	27.45	27.05	26.89	27.38	Low KOC
L141	28.26	27.79	25.39	25.78	25.47	26.52	Low KOC
L142	28.06	29.4	25.2	26.21	26.64	27.18	Low KOC
L149	27.66	29.08	26.17	26.58	28.03	27.63	Low KOC
L151	27.86	29.72	26.13	26.49	27.48	27.65	Low KOC
L153	28.23	29.11	26.27	27.53	27.09	27.66	Low KOC
L154	27.82	27.07	26.08	25.47	26.49	26.62	Low KOC
L155	27.82	27.98	25.73	25.84	26.81	26.9	Low KOC
L160	26.44	28.35	27.02	26.52	25.94	26.88	Low KOC
L161	27.28	27.45	25.84	27.08	28.01	27.19	Low KOC
L163	27.54	NA	26.51	26.75	27.23	27.03	Low KOC
L165	29.82	29.2	25.34	27.57	26.79	27.71	Low KOC
L166	28.76	28.14	25.93	25.83	27.03	27.2	Low KOC
L169	26.45	28.88	24.52	25.21	26.68	26.51	Low KOC
L170	27.39	28.14	26.31	25.38	25.87	26.67	Low KOC
L171	26.2	27.99	25.97	25.4	25.56	26.29	Low KOC
L174	29.14	28.58	26.08	26.83	26.11	27.31	Low KOC
L175	28.04	28.16	26.08	25.8	25.26	26.65	Low KOC
L176	28.07	26.86	26.34	24.94	26.15	26.51	Low KOC
L181	27.59	28.69	26.42	25.42	27.27	27.21	Low KOC
L183	26.73	29.39	26.61	26.57	27.91	27.59	Low KOC
L184	27.18	28.14	26.16	25.61	27.37	27.02	Low KOC
L185	26.62	28.71	25.01	25.56	25.17	26.26	Low KOC
L186	28.42	27.75	24.66	24.67	25.3	26.19	Low KOC
L188	27.57	27.32	24.95	26.39	27.63	26.84	Low KOC
L189	26.8	28.53	25.7	23.88	27.18	26.64	Low KOC
L192	28.66	28.43	26.25	26.67	27.22	27.48	Low KOC
L196	26.91	28	24.22	27.83	26.54	26.68	Low KOC
L197	27.07	27.63	25.16	25.74	27.59	26.76	Low KOC
L200	28.07	26.67	25.42	28.26	26.37	26.83	Low KOC
L201	26.79	27.48	25.54	26	26	26.39	Low KOC
L203	26.56	26.97	25.05	24.84	26.07	25.98	Low KOC
L204	27.5	27.09	27.04	25.24	25.99	26.6	Low KOC
L205	28.72	28.59	26.66	26.3	27.3	27.57	Low KOC
L206	29.22	29.04	26.61	25.94	26.34	27.46	Low KOC

L209	28.48	27.98	26.29	26.56	27.38	27.38	Low KOC
L210	28.05	NA	27.7	25.02	26.16	26.76	Low KOC
L213	27.15	27.22	26.2	26.41	27.54	26.97	Low KOC
L215	27.7	28.23	25.68	25.81	26.11	26.75	Low KOC
L216	26.44	28.53	24.82	23.95	26.59	26.27	Low KOC
L218	28.36	29.37	27.33	26.62	26.34	27.63	Low KOC
L219	26.51	27.88	26.02	25.18	26.78	26.6	Low KOC
L221	27.2	26.26	27.83	24.98	26.88	26.69	Low KOC
L223	28.06	28.06	NA	NA	26.31	27.39	Low KOC
L224	28.79	29.14	27.64	26.12	25.95	27.53	Low KOC
L225	27.27	28.53	26.99	25.3	26.45	27.01	Low KOC
L226	27.92	26.09	27.36	27.29	27.41	27.17	Low KOC
L227	27.59	28.93	26.68	24.14	27.48	27.18	Low KOC
L230	25.77	27.75	28.39	24.53	26.01	26.61	Low KOC
L231	26.96	27.83	27.88	26.39	26.9	27.24	Low KOC
