# Supplementary material for: Hub Genes in Stable QTLs Orchestrate the Accumulation of Cottonseed Oil in Upland Cotton via Catalyzing Key Steps of Lipid-Related Pathways
Source: Int J Mol Sci. 2023 Nov 22;24(23):16595. doi: 10.3390/ijms242316595 (PMC10706765; doi:10.3390/ijms242316595)
Supplement: Supplementary file 1 [file ijms-24-16595-s001.zip › Table S5.pdf]

Table S5 Basic information of KOC QTLs of cottonseed dentified via SLAF-seq strategy

Strategies	QTLs	Env No	Env	Position (cM)	LOD	Additive effect*		R2	References
						♂(+)	♀(-)		
slaf	qOC^{slaf}-c1-1	1	15AY	0.61	2.23	0.78		3.83	
slaf	qOC^{slaf}-c1-2	2	16AY	6.01	2.25	2.63		47.38	
			BLUE	6.01	3.59	2.31		48.35	
slaf	qOC^{slaf}-c1-3	1	15ALE	48.91	2.07		-1.35	4.17	
slaf	qOC^{slaf}-c2-1	1	15AY	154.61	2.60	1.16		19.90	
slaf	qOC^{slaf}-c2-2	1	14KEL	164.21	2.75	0.93		5.08	
slaf	qOC^{slaf}-c2-3	1	14AY	169.21	2.44	0.84		4.97	
slaf	qOC^{slaf}-c4-1	1	14KEL	37.81	2.27		-0.73	4.27	
slaf	qOC^{slaf}-c4-2	3	15AY	91.51	4.12	1.43		25.22	
			16AY	90.51	4.25	1.15		16.78	
			BLUE	90.51	3.02	0.93		16.55	
slaf	qOC^{slaf}-c5-1	1	15ALE	83.41	1.95	0.72		3.78	
slaf	qOC^{slaf}-c5-2	1	14KEL	87.01	2.07	0.59		3.86	
slaf	qOC^{slaf}-c5-3	1	14AY	83.91	3.78	0.65		6.89	
slaf	qOC^{slaf}-c7-1	6	14AY	28.31	2.85		-0.76	5.22	
			14KEL	37.41	2.06		-0.46	3.57	
			15ALE	28.31	2.36		-0.68	4.26	
			15AY	28.31	4.26		-1.04	7.75	
			16AY	28.31	4.79		-1.11	8.91	

			BLUE	28.31	4.36		-0.87	7.89
slaf	qOC^{slaf}-c7-2	2	15AY	38.61	2.63	0.82		5.05
			BLUE	38.91	3.01	0.69		5.73
slaf	qOC^{slaf}-c7-3	4	14AY	48.91	4.15	0.88		7.35
			14KEL	45.91	2.02	0.57		3.20
			15ALE	47.41	2.50	0.59		4.41
			BLUE	48.91	2.84	0.65		4.79
slaf	qOC^{slaf}-c7-4	1	14KEL	78.31	2.61	0.60		4.13
slaf	qOC^{slaf}-c7-5	1	14KEL	112.31	2.36	0.55		4.78
slaf	qOC^{slaf}-c7-6	1	14KEL	119.11	2.60	0.61		4.46
slaf	qOC^{slaf}-c7-7	1	14KEL	162.11	4.04	1.33		6.66
slaf	qOC^{slaf}-c8-1	1	15ALE	35.01	2.69		-0.69	5.04
slaf	qOC^{slaf}-c8-2	1	15ALE	40.41	2.09		-0.91	3.93
slaf	qOC^{slaf}-c8-3	1	15ALE	66.91	2.30	0.89		4.21
slaf	qOC^{slaf}-c8-4	1	14KEL	125.21	2.50	0.74		9.59
slaf	qOC^{slaf}-c9-1	1	14AY	22.41	2.18		-0.36	4.00
slaf	qOC^{slaf}-c10-1	2	14AY	7.01	2.75	0.65		5.56
			16AY	7.01	2.79	0.75		5.58
slaf	qOC^{slaf}-c10-2	2	14AY	18.01	1.95		-0.52	3.65
			15AY	18.01	2.14		-0.62	3.99
slaf	qOC^{slaf}-c11-1	1	15AY	12.91	2.15	0.66		4.49
slaf	qOC^{slaf}-c12-1	1	15AY	10.01	2.07	0.44		4.38

slaf	qOC^{slaf}-c12-2	1	16AY	18.91	2.08	0.51		3.73
slaf	qOC^{slaf}-c13-1	1	14AY	17.11	6.66	1.13		11.68
slaf	qOC^{slaf}-c13-2	1	15ALE	20.01	4.01	0.85		7.51
slaf	qOC^{slaf}-c13-3	3	14KEL	21.31	6.38	1.29		11.90
			15AY	21.31	4.17	0.98		7.74
			BLUE	21.31	4.84	0.88		9.05
slaf	qOC^{slaf}-c13-4	4	14AY	27.21	5.79	1.10		10.24
			15ALE	27.21	2.00	0.69		3.55
			15AY	27.21	3.47	1.19		6.05
			BLUE	27.21	4.12	1.08		7.23
slaf	qOC^{slaf}-c15-1	2	14KEL	4.51	8.51	3.13		51.95
			15ALE	4.51	2.80	1.85		41.14
slaf	qOC^{slaf}-c15-2	4	14KEL	63.91	3.19		-2.82	14.18
			15ALE	60.11	2.94		-0.70	7.20
			15AY	62.11	2.69		-0.54	5.34
			16AY	62.11	2.27		-0.51	4.57
slaf	qOC^{slaf}-c14-1	1	16AY	49.01	2.13	1.57		4.16
slaf	qOC^{slaf}-c14-2	1	15AY	126.91	1.98		-0.71	4.34
slaf	qOC^{slaf}-c17-1	1	15AY	49.21	3.80		-1.99	17.45
slaf	qOC^{slaf}-c17-2	5	14KEL	138.61	9.50	3.04		53.24
			15ALE	138.61	4.12	2.02		47.86
			15AY	138.61	6.80	2.76		47.44

			16AY	138.61	3.08	3.09	46.80	
			BLUE	138.61	10.75	2.77	52.07	
slaf	qOC^{slaf}-c17-3	6	14AY	148.21	10.08	2.42	46.57	(Du et al., 2018; Zhao et al., 2019; Zhu et al., 2020; Gong et al., 2022)
			14KEL	147.21	14.49	3.23	55.28	
			15ALE	147.21	5.80	2.21	47.79	
			15AY	148.21	14.00	2.76	53.40	
			16AY	148.21	11.97	3.33	54.75	
			BLUE	148.21	20.62	2.83	59.04	
slaf	qOC^{slaf}-c17-4	5	14AY	154.11	4.35	2.41	43.09	
			14KEL	154.11	5.17	3.21	53.07	
			15AY	155.11	7.65	2.85	48.75	
			16AY	155.11	5.97	3.10	51.15	
			BLUE	155.11	14.11	2.90	58.83	
slaf	qOC^{slaf}-c17-5	5	14AY	162.41	4.76	2.34	43.97	
			14KEL	163.41	8.74	3.13	54.06	
			15AY	162.41	6.90	2.68	44.57	
			16AY	162.41	5.31	3.14	48.07	
			BLUE	162.41	12.68	2.95	54.28	
slaf	qOC^{slaf}-c19-1	1	15AY	88.91	2.13	0.64	3.95	
slaf	qOC^{slaf}-c19-2	3	14AY	140.01	3.36	0.84	7.78	
			15AY	138.01	3.24	0.97	9.68	
			16AY	138.01	2.17	0.71	4.90	

slaf	qOC^{slaf}-c25-1	1	16AY	52.21	2.23	2.26	18.74	
slaf	qOC^{slaf}-c25-2	1	14KEL	117.11	4.18	2.78	50.61	
slaf	qOC^{slaf}-c25-3	6	15ALE	121.81	3.24	1.72	11.39	
			14AY	122.81	5.50	3.11	38.69	
			15AY	122.81	7.86	2.93	35.17	
			16AY	122.81	9.49	3.65	43.09	
			14KEL	122.81	10.02	3.21	43.01	
			BLUE	122.81	13.55	3.12	47.03	
slaf	qOC^{slaf}-c24-1	1	14KEL	60.21	2.06	1.02	4.41	
slaf	qOC^{slaf}-c24-2	5	14KEL	89.81	18.00	3.30	60.50	
			15ALE	89.81	2.90	2.34	41.42	
			15AY	88.81	12.74	3.29	56.63	
			16AY	89.81	12.93	3.53	51.25	
			BLUE	89.81	20.32	2.98	59.79	
slaf	qOC^{slaf}-c24-3	6	14AY	96.21	7.72	2.86	47.64	
			14KEL	95.21	17.01	3.21	55.90	
			15ALE	98.21	3.71	1.77	46.52	
			15AY	95.21	12.98	3.29	54.64	
			16AY	95.21	13.58	3.38	53.35	
			BLUE	95.21	19.95	2.92	57.82	
slaf	qOC^{slaf}-c23-1	4	14KEL	30.01	2.97	0.52	5.34	(Gong et al., 2022)
			15ALE	42.91	2.29	0.40	4.18	

			15AY	42.91	3.50	0.55		6.44
			BLUE	41.21	3.36	0.44		6.13
slaf	qOC^{slaf}-c23-2	3	15AY	66.81	1.96		-0.41	3.54
			16AY	66.81	2.10		-0.43	3.67
			BLUE	66.81	2.30		-0.37	3.99
slaf	qOC^{slaf}-c20-1	1	16AY	19.91	3.05		-0.71	5.42
slaf	qOC^{slaf}-c20-2	4	15ALE	113.71	2.33	1.14		4.31
			15AY	112.71	3.21	2.04		15.87
			16AY	112.71	2.69	1.53		7.02
			BLUE	112.71	2.60	1.36		8.52
slaf	qOC^{slaf}-c26-1	3	14AY	84.81	2.04		-0.93	3.66
			15AY	85.81	2.96		-1.58	7.70
			16AY	84.81	2.37		-1.13	4.11
slaf	qOC^{slaf}-c26-2	4	14AY	96.71	3.45	1.19		6.76
			15AY	96.71	2.05	1.02		3.81
			14KEL	96.71	2.86	1.26		5.17
			BLUE	96.71	3.33	1.09		6.33
slaf	qOC^{slaf}-c18-1	1	15ALE	19.91	1.86	0.52		3.54
slaf	qOC^{slaf}-c18-2	1	14KEL	116.61	1.74	1.47		4.13

*♂ represents paternal XLZ24, and ♀ represents maternal LMY28.
