# Supplementary material for: Hub Genes in Stable QTLs Orchestrate the Accumulation of Cottonseed Oil in Upland Cotton via Catalyzing Key Steps of Lipid-Related Pathways
Source: Int J Mol Sci. 2023 Nov 22;24(23):16595. doi: 10.3390/ijms242316595 (PMC10706765; doi:10.3390/ijms242316595)
Supplement: Supplementary file 1 [file ijms-24-16595-s001.zip › Table S6.pdf]

Table S6 Basic information of KOC QTLs of cottonseed identified via CHIP strategy

Strategies	QTLs	Env No	Env	Position (cM)	LOD	Additive effect* ♂(+)	Additive effect* ♀(-)	R2(%)	References
chip	qOC^{chip}-c1-1	4	14AY	20.41	2.59	0.45		4.89	
			14KEL	19.41	2.19	0.52		4.15	
			15ALE	16.81	2.06	0.37		4.01	
			BLUE	20.41	2.10	0.37		4.25	
chip	qOC^{chip}-c1-2	1	14AY	31.61	3.39	0.63		7.01	
chip	qOC^{chip}-c3-1	1	15AY	48.61	2.08	0.46		3.87	
chip	qOC^{chip}-c3-2	2	14KEL	57.81	2.65	0.59		5.03	
			16AY	55.41	2.01	0.47		3.66	
chip	qOC^{chip}-c3-3	1	15AY	66.61	2.46		-0.49	4.35	
chip	qOC^{chip}-c4-1	1	14AY	85.71	2.13		-0.37	4.02	
chip	qOC^{chip}-c5-1	1	BLUE	8.81	2.54		-0.43	5.80	
chip	qOC^{chip}-c5-2	2	16AY	77.31	2.76	0.46		4.77	
			BLUE	77.31	2.08	0.33		3.63	
chip	qOC^{chip}-c5-3	3	15ALE	149.31	2.27	0.36		3.88	(Gong et al., 2022)
			15AY	150.31	2.83	0.49		5.23	
			BLUE	150.21	1.69	0.36		2.99	
chip	qOC^{chip}-c6-1	3	14AY	51.21	5.47	0.63		10.22	(Gong et al., 2022)
			15AY	51.71	2.92	0.49		5.64	
			BLUE	51.11	2.54	0.38		4.88	

chip	qOC^{chip}-c6-2	2	14KEL	58.41	1.73	0.58		3.34
			BLUE	56.41	2.33	0.41		5.52
chip	qOC^{chip}-c7-1	1	BLUE	44.41	2.77		-2.06	5.18
chip	qOC^{chip}-c7-2	1	15AY	69.61	2.32		-1.71	4.59
chip	qOC^{chip}-c7-3	1	14AY	128.31	2.14		-1.82	5.88
chip	qOC^{chip}-c8-1	1	15ALE	11.41	4.02	0.70		7.02
chip	qOC^{chip}-c8-2	1	15ALE	32.11	1.86		-0.44	3.44
chip	qOC^{chip}-c9-1	1	14AY	34.81	2.60		-0.40	4.70
chip	qOC^{chip}-c9-2	1	14KEL	47.41	2.63	0.45		4.27
chip	qOC^{chip}-c10-1	2	14KEL	8.21	2.03	0.54		5.76
			16AY	11.21	3.17	0.60		8.20
chip	qOC^{chip}-c10-2	2	14AY	34.41	2.17	0.48		4.19
			BLUE	34.41	2.41	0.47		4.43
chip	qOC^{chip}-c10-3	1	14KEL	38.11	2.38	0.46		4.00
chip	qOC^{chip}-c10-4	1	16AY	64.51	2.88		-0.60	5.35
chip	qOC^{chip}-c10-5	1	16AY	67.11	2.22		-0.54	4.13
chip	qOC^{chip}-c10-5	3	14KEL	78.21	4.08	0.65		7.47
			15ALE	78.41	4.26	0.54		7.51
			16AY	78.21	3.85	0.68		7.41
chip	qOC^{chip}-c11-1	1	14AY	35.41	2.30	0.56		4.39
chip	qOC^{chip}-c12-1	2	14KEL	41.31	4.20	0.73		7.04
			BLUE	41.31	2.56	0.43		4.15

chip	qOC^{chip}-c12-2	1	15ALE	62.11	2.08	0.45		3.52
chip	qOC^{chip}-c12-3	5	14KEL	122.21	2.19		-0.46	3.89
			15ALE	122.21	2.55		-0.40	4.66
			15AY	122.21	2.04		-0.40	3.61
			16AY	122.21	3.05		-0.50	5.45
			BLUE	122.21	2.96		-0.41	5.44
chip	qOC^{chip}-c13-1	2	14KEL	1.51	2.24	0.75		5.11
			15ALE	1.51	2.38	0.64		5.53
chip	qOC^{chip}-c13-2	1	16AY	42.01	2.31		-0.45	4.17
chip	qOC^{chip}-c13-3	1	14KEL	78.61	2.35	0.45		4.38
chip	qOC^{chip}-c13-4	2	15AY	102.41	3.05	0.47		5.35
			BLUE	102.41	2.54	0.35		4.41
chip	qOC^{chip}-c14-1	2	15AY	71.21	2.43	0.69		4.38
			BLUE	70.71	2.19	0.55		3.93
chip	qOC^{chip}-c14-2	1	14AY	75.71	1.75	0.48		3.13
chip	qOC^{chip}-c14-3	1	16AY	106.61	2.12		-0.48	3.99
chip	qOC^{chip}-c16-1	1	15ALE	26.91	2.04	0.38		4.52
chip	qOC^{chip}-c17-1	1	15ALE	4.61	1.71	0.33		3.29
chip	qOC^{chip}-c18-1	1	15ALE	22.51	2.50	0.41		4.75
chip	qOC^{chip}-c19-1	2	16AY	25.61	3.03		-0.49	5.57
			BLUE	25.61	2.63		-0.37	4.79
chip	qOC^{chip}-c19-2	1	14AY	68.01	1.92	0.43		3.25

chip	qOC^{chip}-c19-3	1	BLUE	92.61	2.16		-0.39	4.51	
chip	qOC^{chip}-c20-1	3	15ALE	32.11	3.69	0.54		6.66	
chip	qOC^{chip}-c21-1	3	14KEL	19.91	2.69	0.55		5.11	
			15AY	16.31	2.49	0.71		7.96	
			BLUE	16.31	3.30	0.69		11.10	
chip	qOC^{chip}-c21-2	3	14AY	32.11	2.71		-0.61	6.86	
			14KEL	35.11	3.60		-0.88	10.49	
			BLUE	33.11	2.15		-0.54	6.43	
chip	qOC^{chip}-c23-1	6	14AY	24.71	3.22	0.43		5.58	(Gong et al., 2022)
			14KEL	25.41	2.01	0.40		3.36	
			15ALE	25.41	2.45	0.38		4.43	
			15AY	25.51	3.34	0.55		6.26	
			16AY	25.51	2.13	0.43		3.98	
			BLUE	25.51	4.00	0.46		6.97	
chip	qOC^{chip}-c23-2	1	14AY	30.41	1.97	0.47		5.82	
chip	qOC^{chip}-c25-1	3	14KEL	83.91	3.14	0.60		5.40	
			15ALE	83.91	2.12	0.41		3.90	
			BLUE	83.91	2.07	0.39		3.80	
chip	qOC^{chip}-c26-1	2	14KEL	37.61	2.66	0.53		5.08	
			16AY	36.61	2.83	0.46		4.81	

*♂ represents paternal XLZ24, and ♀ represents maternal LMY28.
